# Non-canonical *Staphylococcus aureus* pathogenicity island repression

**DOI:** 10.1093/nar/gkac855

**Published:** 2022-10-06

**Authors:** Laura Miguel-Romero, Mohammed Alqasmi, Julio Bacarizo, Jason A Tan, Richard J Cogdell, John Chen, Olwyn Byron, Gail E Christie, Alberto Marina, José R Penadés

**Affiliations:** MRC Centre for Molecular Bacteriology and Infection, Imperial College London, SW7 2AZ, UK; Institute of Infection, Immunity and Inflammation, University of Glasgow, Glasgow, G12 8TA, UK; Institute of Infection, Immunity and Inflammation, University of Glasgow, Glasgow, G12 8TA, UK; College of Applied Medical Sciences, Shaqra University, Shaqra City 15572, Saudi Arabia; MRC Centre for Molecular Bacteriology and Infection, Imperial College London, SW7 2AZ, UK; Departamento de Ciencias Biomédicas, Universidad CEU Cardenal Herrera, 46113 Moncada, Spain; Department of Microbiology and Immunology, Virginia Commonwealth University, Richmond, VA 23298, USA; School of Molecular Biosciences, University of Glasgow, G12 8QQ,UK; Infectious Diseases Translational Research Programme and Department of Microbiology and Immunology, Yong Loo Lin School of Medicine, National University of Singapore, 5 Science Drive 2, Singapore; School of Life Sciences, University of Glasgow, Glasgow, G12 8QQ,UK; Department of Microbiology and Immunology, Virginia Commonwealth University, Richmond, VA 23298, USA; Instituto de Biomedicina de Valencia (IBV), CSIC and CIBER de Enfermedades Raras (CIBERER), Valencia, Spain; MRC Centre for Molecular Bacteriology and Infection, Imperial College London, SW7 2AZ, UK

## Abstract

Mobile genetic elements control their life cycles by the expression of a master repressor, whose function must be disabled to allow the spread of these elements in nature. Here, we describe an unprecedented repression-derepression mechanism involved in the transfer of *Staphylococcus aureus* pathogenicity islands (SaPIs). Contrary to the classical phage and SaPI repressors, which are dimers, the SaPI1 repressor Stl^SaPI1^ presents a unique tetrameric conformation never seen before. Importantly, not just one but two tetramers are required for SaPI1 repression, which increases the novelty of the system. To derepress SaPI1, the phage-encoded protein Sri binds to and induces a conformational change in the DNA binding domains of Stl^SaPI1^, preventing the binding of the repressor to its cognate Stl^SaPI1^ sites. Finally, our findings demonstrate that this system is not exclusive to SaPI1 but widespread in nature. Overall, our results characterize a novel repression-induction system involved in the transfer of MGE-encoded virulence factors in nature.

## INTRODUCTION

*Staphylococcus aureus* pathogenicity islands (SaPIs) are prototypical members of a widespread family of mobile genetic elements (MGEs), the phage-inducible chromosomal islands (PICIs) (1). SaPIs are clinically important because they encode and disseminate toxin and antibiotic resistance genes (1,2). Normally, these elements reside passively integrated in host bacterial chromosomes under the control of a master repressor protein, Stl (SaPI transcription leftward) (3). Unlike most phage repressors, SaPI Stl repressors are not cleaved after induction of the cellular SOS response. This is because the Stl repressors are insensitive to the activated RecA protein. Instead, SaPI activation depends on the formation of a complex between Stl and specific phage proteins, which act as inducers of the SaPI life cycle (4–6). Since SaPIs require the phage components for DNA packaging and particle assembly (7,8), this strategy ensures that SaPIs will be induced only in the presence of their prey, the phages.

Different SaPIs encode different repressors and therefore require different phage proteins as inducers. Thus, the inducers for SaPIbov1, SaPI1 or SaPI2 are the phage-encoded dUTPases, Sri or recombinase proteins, respectively (4–6). To gain more insight into this interesting and distinctive induction-repression mechanism of the SaPIs, we recently solved the structure of the SaPIbov1 Stl (Stl^SaPIbov1^) repressor alone and complexed with two different inducing proteins: the dimeric and trimeric dUTPase proteins of phages O11 and ϕ11, respectively (9). Our studies revealed that Stl^SaPIbov1^ is a canonical dimer, with a modular structural organization reminiscent of many well-studied phage and MGE repressors, including the CI repressor of archetypical phage λ (9,10). These repressors have N-terminal domains that recognize and bind to their cognate DNA operator regions, and C-terminal domains that are involved in repressor dimerization and inducer recognition. Phages and other MGEs that are activated by the SOS response encode repressors that are structural homologs of LexA and are induced to undergo self-cleavage by activated RecA*. Since repressor dimers are required for repression, self-cleavage disrupts dimerization and activates the life cycle of prophages and other MGEs (11,12). In the case of SaPIbov1, Stl^SaPIbov1^ forms dimers that are disrupted upon binding to their cognate phage inducers (9).

Our previous findings suggested that the general mechanism for the inactivation of Stl^SaPIbov1^ and the classical CI-like repressors was disruption of their dimerization, though the ways by which this occurred were different. In the case of Stl^SaPIbov1^, a domain with the dual role of mediating dimerization and binding inducer enables the SaPIbov1 island to sense the activation of a helper phage. However, the existence of multiple different Stl repressor proteins raises the question of whether the Stl^SaPIbov1^ mechanism of inactivation is conserved across all SaPI repressors or whether they sense helper phages via other unknown strategies. To answer this question, we analysed SaPI1, which is one of the prototypical islands used to decipher the biology of the SaPIs. It is also clinically relevant because it encodes TSST-1, the toxin responsible for a rare but important human disease known as toxic shock syndrome (13). Two additional factors reinforced the use of SaPI1 as a model. Firstly, Sri, its anti-repressor protein (5), is a small protein of 6.2 kDa, which raises the question of how this small protein de-represses SaPI1. Secondly, and contrary to what is seen with SaPIbov1 and other prophages, our unpublished results indicate that the repression mediated by the SaPI1 Stl (Stl^SaPI1^) is extremely strong. Thus, while some SaPIs—such as SaPIbov1 or SaPIbov2—exhibit some level of basal excision from the bacterial chromosome in the absence of helper phage (14), SaPI1 remains integrated, suggesting that this island has a different repression system. Here we report the discovery of a new repression-derepression system involved in the control and transfer of MGEs. Importantly, our results demonstrate that this new system is not exclusive to the SaPIs but is utilized by other MGEs in nature.

## MATERIALS AND METHODS

### Bacterial strains and growth conditions

Bacterial strains used in this study are listed in Supplementary Table S1. Strains were grown at 37°C in Luria-Bertani broth agar or in Luria-Bertani broth with shaking (180 rpm) for *Escherichia coli*, or in tryptic soy (TSA) agar or TSB broth for *S. aureus*. Ampicillin (100 mg/ml) or tetracycline (20 mg/ml; all Sigma-Aldrich) antibiotics were added when appropriate.

### Plasmid construction

The plasmids used in this study (Supplementary Table S2) were constructed by cloning with T4 ligase (Thermofisher) the PCR products, amplified with the oligonucleotides listed in Supplementary Table S3 (Sigma-Aldrich), into the appropriate vectors after their digestion with restriction enzymes. The cloned plasmids were verified by Sanger sequencing (Eurofins Genomics). Stl^SaPI1^ (Uniprot Accession code O54475) and 80α phage Sri (Uniprot Accession code A4ZF88) proteins were cloned into the pPROEX Hta plasmid. For Stl^SaPI1^ expression alone, a His^6^-tag and TEV protease cleavage site were added to the N-terminal part of the protein. For the Sri-Stl^SaPI1^ complex expression, both proteins were cloned into plasmid pPROEX Hta, under the control of the Trc IPTG inducible promoter, but only the Sri protein had the His^6^-tag and the TEV protease cleavage site fused to its N-terminal region. In this plasmid expressing both proteins, an extra ribosomal binding site was added upstream to the SaPI1 *stl* gene (3’ of the *sri* gene). For the *in vivo* experiments, the SaPI1 region between *int* and *xis*, which includes the *entK, entQ, stl* and *str* genes and the putative Stl binding sites present in the intergenic region between *stl* and *str*, was cloned into the plasmids pCN41 with the β-lactamase gen fused to *xis*.

### Protein expression and purification

Proteins were overexpressed from *E. coli* BL21(DE3) (Novagen) cells transformed with the corresponding expression plasmids (Supplementary Table S2). Cultures were grown in Luria-Bertani broth to an OD_600_ of 0.5–0.6 and protein expression was induced with 1 mM IPTG at 20ºC for 16 h. Cells were harvested by centrifugation at 4ºC, 4000 rpm for 20 min, resuspended in lysis buffer (100 mM Tris pH 8, 300 mM NaCl, 5ml Mg_2_Cl, 1mM β-mercaptoethanol and protease inhibitor tablets (complete tablets, Roche) and lysed by sonication. The soluble fractions were obtained by centrifugation at 4ºC, 15 000 rpm for 30 min and loaded onto a pre-equilibrated Nickel affinity column (Histrap 1 ml; GEHealthcare). After washing the column with a buffer with 20 mM imidazole, the proteins were eluted with a lysis buffer containing 300 mM imidazole. The fractions containing the eluted proteins were further purified by size exclusion chromatography using a Superdex S200 16/600 column and analysed by SDS-PAGE. Fractions containing the purest protein were selected, concentrated to 8–10 mg/ml and stored at –80ºC. For protein crystallization, the Sri-Stl^SaPI1^ selenomethionine-labeled (SeMet) derivative complex was obtained using SelenoMethionine Medium Complete (Molecular Dimensions Ltd; MD 12–500), according to the manufacturer instructions, and purified as described previously. The Sri-Stl^SaPI1^ and Sri-Stl^SaPI1 L201E^ complexes used for crystallization, SEC-MALS and biolayer interferometry were digested with the TEV protease to eliminate the His^6^-tag at 4ºC overnight in 100 mM Tris–HCl pH8, 300 mM NaCl and 5 mM Mg_2_Cl buffer before further purification by size exclusion.

### Protein crystallization and data collection

Crystals of 80α Sri-Stl^SaPI1^ SeMet derivative and 80α Sri-Stl^SaPI1 L201E^ complexes were obtained by vapor-diffusion technique using a sitting drop setup at 15ºC in a reservoir solution of 0.4 M ammoniun phosphate, 25% PEG200 or 0.1 M Tris–HCl pH 8.5, 22% PEG350, respectively. The crystals were cryo-protected using 30% PEG200 or PEG350 solution for Sri-Stl^SaPI1^ or Sri-Stl^SaPI1 L201E^ respectively when freezing in liquid nitrogen. Sri-Stl^SaPI1^ single-wavelength anomalous diffraction (SAD) was undertaken on beamline I03 at the Diamond Light Source synchrotron radiation facility (DLS; Didcot, UK) (15) at a wavelength of 0.98 Å. The Sri-Stl^SaPI1 L201E^ native dataset was collected on beamline I04-1 at DLS at a wavelength of 0.915 Å. For Sri-Stl^SaPI1^ SeMet, data were indexed, integrated, and anisotropically scaled using the program DIALS and phasing was performed with CRANK2 both from CCP4 suite (17). The anomalous map for Sri-Stl^SaPI1^ data set was obtained with Phenix suite and the peak intensities at the coordinates of selenium atoms in methionine residues are collected in Supplementary Table S4. For Sri-Stl^SaPI1 L201E^, data were indexed, integrated, and scaled using the program AutoProc (16) and the phasing was performed by Molecular Replacement with Phaser (17) using the monomer of wt Sri-Stl^SaPI1^ complex as a search model. Final models were generated by several rounds of manual model building using Coot (18) and computational refinement with Refmac5 (17). The crystallographic parameters, data-collection and refinement statistics are listed in Table 1.

**Table 1.** Data collection and refinement statistics. Data collection and refinement statistics for Sri-Stl^SaPI1^ and Sri-Stl^SaPI1 L201E^ X-ray structures. PDB, protein data bank. Highest-resolution shell details are shown in parenthesis

	80α Sri-Stl^SaPI1^	80α Sri-Stl^SaPI1 L201E^
***Data collection***		
Space group	*P*6_1_22	*P*3_2_21
Cell dimensions *a, b, c* (Å)	100.21, 100.21, 298.69	88.22, 88.22, 110.49
α, β, γ (°)	90, 90, 120	90, 90, 120
Wavelength (Å)	0.979	0.916
Resolution	86.79–2.90 (3.08–2.9)	62.84–2.97 (3.17–2.97)
*R*_merge_ (%)	0.072 (0.514)	0.054 (0.589)
Mean *I/σ* (I)	14.2 (2.4)	12.3 (1.3)
Completeness (%)	99.7 (99.5)	92.0 (46.3)
Multiplicity	5.9 (5.8)	9.8 (9.3)
*R*_pim_	0.041 (0.246)	0.046 (0.629)
CC1/2	0.845 (0.951)	0.999 (0.493)
***Refinement***		
Resolution (Å)	83.48–2.90	62.84–2.97
No. of reflections (observed/unique)	121 050/20 492 (18 792/3215)	87 460/8 900 (4129/444)
		
*R*_work_/*R*_free_ (%)	0.30/0.34	0.25/0.29
Number of atoms:		
Protein	4208	2435
Water	8	2
***B-factors (* *Å* *)***		
Protein	104.87	104.38
Water	73.8	57.99
***r.m.s* *deviation***		
Bond lengths (Å)	0.0050	0.0018
Bond angles (°)	1.32	1.15
***PDB code***	7P4A	1ZVI

### Size-exclusion chromatography multi-angle light scattering (SEC-MALS)

The wt and L201E Stl^SaPI1^ proteins at 2 mg/ml alone or in complex with Sri in 50 mM Tris–HCl pH 7.5, 250 mM NaCl, 5 mM MgCl_2_ were loaded in a Protein KW403 column (Shodex) equilibrated with the same buffer using a HPLC (Shimadzu) system. Chromatography was run at 0.4 ml/min and the UV, light scattering and differential refractive index (dRI) was monitored using a TREOS (Wyatt) system. The data collection and analysis were performed using ASTRA 7.3.2.21 software. The UV and MW representation was done by Excel.

### Characterization of the SaPI1 *str* and *stl* promoters

To characterize the *str* and *stl* promoters, RNA extraction using an Ambion kit (Novartis) was performed according to the manufacturer instructions using the RN4220 strain lysogenic for 80⍺ carrying SaPI1, 90 min after prophage induction with 2 μg/ml mitomycin C (MC). A 5′/3′RACE Kit (Roche) was used to amplify the RNA obtained and the final DNA was sequenced to obtain the transcription start site nucleotide of both promoters. The primers used are summarised in the Supplementary Table S5. The –10 and –35 RNA pol binding sites were localized after the analysis of the DNA sequence.

### β-Lactamase assays

Cells were obtained at different time points after mitomycin C (MC) induction of lysogenic strains carrying the appropriate plasmids. β-lactamase assays, using nitrocefin as substrate, were performed as described (4,6). Briefly, 50 μl of the collected sample were mixed with 50 μl of nitrocefin stock solution (192 μM made in 50 mM potassium phosphate buffer, pH 5.9), and the absorbance at 490 nm immediately read using a FLUOstar Omega microplate reader (BMG LABTECH) for 45 min. Promoter activity was calculated as Promoter activity = $\frac{{( {\frac{{d{A_{490}}}}{{dt}}} )}}{{{A_{540}}dV}}$, where A_540_ is the absorbance of the sample at 540 nm at collection, t is time, d is the dilution factor, and V is the sample volume.

### End-labeling SaPI1 *stl-str* DNA for footprinting experiments

SaPI1 *stl-str* DNA was amplified using *PfuTurbo* DNA polymerase (Agilent), dNTPs (Invitrogen), and primers GC83016C (5’-GTTCTTTAACCGAAAGCTTCCAACTCACTCTTTC-3’) and GC83016B (5’-CAGTACGTTCGTGATACAAGCCATGTATTGATGTTC-3’). The PCR product was diluted 1:1,000 (approximately 0.001 pmol/μl) for preparing radiolabeled SaPI1 *stl-str* DNA. 5 pmol of primer (5 μl of 5 μM stocks) were end-labelled with 10 units of USB Optikinase (Affymetrix), and 7 μl of 6000Cu/mmol ATP-ɣ32P (Perkin-Elmer) in 25 μl total volume. Incorporation of ATP-ɣ32P into primer was determined by TCA precipitation of a 5 μl aliquot of a 1:200 dilution and counting the precipitated material on filter paper using a scintillation counter. To obtain final radiolabelled SaPI1 *stl-str* DNA (*) used in footprint experiments, the remaining 24.5 μl of the undiluted Optikinase reaction (containing ∼1 pmol/5 μl of either top- or bottom-strand labelled primer) was added to the original 1:1000 dilution of the SaPI1 PCR product (template), 1 μl of 10 mM dNTP (Invitrogen), and 1 μl of *PfuTurbo* DNA polymerase (Agilent) for amplification in a final volume of 50 μl. Unincorporated primers were removed using a QIAquick PCR Purification Kit (Qiagen) and eluted twice with 30 μl of water. To assess the final concentration of SaPI1 *stl-str* DNA*, a 1:200 dilution of the PCR clean-up material was made and dpm assessed using the same TCA precipitation/filter method and scintillation counter.

### G/A ladder of SaPI1 *stl-str* DNA

The G/A ladder was generated using the piperidine method. First, 12 μl of SaPI1 *stl-str* DNA* was added to 2 μl of 1 mg/ml salmon sperm DNA and 8 μl of tris-EDTA (TE), pH 8 and incubated on ice. Second, 2 μl of 4% formic acid made fresh from stock was added and incubated at 37°C for 45 min, and subsequently placed back on ice. Third, 300 μl of piperidine (Sigma-Aldrich) was added and incubated at 90°C for 30 min, and subsequently placed back on ice. Fourth, 10 μl of 5 mg/ml salmon sperm DNA (50 mg total) was added followed by gentle mixing. Fifth, 1 ml of butanol was added, mixed, and centrifuged at ∼20 000 rcf (g) for five min; the top layer was carefully removed. A further 1.2 ml of butanol was added, mixed, and centrifuged resulting in a pellet that contained precipitated DNA. Supernatant was removed and the pellet was gently washed with 150 μl of 1% SDS, followed by butanol precipitation (one 1 ml, and two 0.5 ml butanol precipitations). The DNA pellet was dehydrated with a speed vacuum, and resuspended in 20 μl of footprint loading buffer (0.5 ml deionized formamide; 20 μl of 0.25M EDTA, pH 7; 5 μl XCFF and 5 μl of BPB for visualization of sample migration during electrophoresis) and stored at –20°C. Lastly, 1:7.5 and 1:15 dilutions of the G/A ladder were made using the same footprint loading buffer in order to obtain exposure intensities more suitable for sequence determination of the Stl footprints on SaPI1 *stl-str* DNA.

### DNase I footprinting

Footprinting reactions were carried out in EMSA buffer (PBS, pH 7.3, 75 mM NaCl, 5 mM MgCl_2_,1 mM DTT, 0.1 mg/ml BSA, 5% glycerol) supplemented with 2mM CaCl_2_, which is required for DnaseI activity, and 100 ng/ μl poly(d[I-C]) (Sigma-Aldrich), which helps reduce non-specific protein interactions with nucleic acids. The final volume of each footprint reaction was 11 μl. First, a master mix of SaPI1 *stl-str* DNA was made as follows (per 10 total reactions); 10 μl of 0.5 pmol/μl SaPI1 *stl-str* DNA, 10 μl of 1 μg/μl poly(d[I-C]), 20 μl of 5× EMSA buffer, and 2 μl of 100 mM CaCl_2_. 4 μl of SaPI1 *stl-str* DNA master mix was aliquoted into individual tubes on ice. 6 μl of ∼3.3× Stl and/or Sri dilutions were made in EMSA buffer, pre-incubated on ice for 30 min, then added to 4 μl of SaPI1 *stl-str* DNA master mix, and allowed to incubate on ice for a further 30 min. To initiate DNase digestion, 1 μl of DNaseI (2 U/μl) was added to individual reactions and immediately transferred to a 30°C water bath for 10 min. Reactions were quenched by adding 17.2 μl of footprint loading buffer (0.5 mL deionized formamide; 20 μl of 0.25 M EDTA, pH 7; 5 μl XCFF and 5 μl of BPB), followed by immediate transfer to dry ice. Samples were boiled at 100°C for 2 min immediately before loading onto a denaturing gel (5% polyacrylamide, 7 M urea). To help to increase the resolution of the gel electrophoresis, denaturing gels were pre-electrophoresed in 0.5× TBE before adding samples (500 V/400 mA/400 W for 30 min, then 750 V/400 mA/400 W for 30 min; then 1000 V/400 mA/400 W for 30 min and 1250 V/400 mA/400 W for >30 min). 15 μl of the quenched footprint reactions and the G/A ladder dilutions (discussed above) were loaded onto gels. After samples were added to the pre-electrophoresed gel, electrophoresis was continued for ∼3500 Vh for optimal resolution of the Stl-bound regions in SaPI1 *stl-str* DNA. G + A sequencing reactions were performed on the same end-labeled fragments using the method of Maxam and Gilbert (19) and run on the same gel to identify the sequences protected by Stl.

### Electrophoretic mobility shift assay (EMSA)

The Stl^SaPI1^ DNA binding regions for the EMSA experiments were obtained commercially and hybridized at 95ºC for 15 min with equal concentrations of the forward and reverse primers (Supplementary Table S6). DNA at a final concentration of 1 μM and increasing concentrations of the wt or the L201E mutant Stl^SaPI1^ proteins (0.5–8 μM) were mixed with 1 μg/ml poly(d[I-C]) (Roche) in EMSA buffer (50 mM Tris–HCl pH 8, 5 mM MgCl_2_, 1 mM DTT, 0.1 mM EDTA and 5% glycerol) and incubated for 30 min at room temperature. The samples were then loaded onto 6% Tris–borate–EDTA (TBE) polyacrylamide gels and were electrophoresed in TBE buffer at 90 V for 1–2 h. Gels were stained with Gel Red (Biotium) for 10 min in shaking conditions and analysed with a ChemiDoc imaging system (BioRad).

### Biolayer interferometry

The DNA binding kinetics assays with Stl^SaPI1^ and Stl^SaPI1 L201E^ were performed by Biolayer Interferometry with an Octet RED96s System (Sartorius) in 50 mM Tris–HCl pH 8, 300 mM NaCl, 5mM MgCl_2_, 50 μM EDTA pH 8, 0.005% TWEEN at 25ºC and agitation speed of 1400 rpm. The 5′ biotinylated DNA probes, see Supplementary Table S6 for primers sequences, were immobilized at 1.562 μg/ml on previously hydrated streptavidin (SA) Biosensors (Sartorius) for 120 s, reaching a signal of ∼2 nm. After DNA loading, the sensors were washed for 60 s in buffer for a stable baseline and protein association was monitored for 160 s using protein concentrations in a range between 1.562 and 100 nM. The protein concentration range was obtained by 1:2 serial dilutions of the initial stock. After that, dissociation in the same buffer was monitored for 200 s. An empty sensor was used as signal drift control and such signal was subtracted from the obtained curves. The association and dissociation constants (*k*_on_ and *k*_off_) were obtained by fitting a 1:1 model for Stl^SaPI1 L201E^ and a mass transport model for Stl^SaPI1^ in Octet BLI Discovery 12.2.2.20 software.

### Pull-down experiments

The wild type (wt) and the Y76A versions of the Stl^SaPI1^-Sri complex were expressed in 20 ml of culture as previously indicated. The cells where then lysed with BugBuster protein extraction reagent (Novagen) for 30 min at room temperature. The soluble fraction was then incubated for 1 h with HisPur Ni-NTA resin (Thermofisher), and the proteins bound to the resin purified as described before. After purification, the fractions were analysed by SDS-PAGE, stained with Instant Blue (Expedeon) and visualized with a ChemiDoc imaging system (BioRad).

### SaPI transfer

*S. aureus* strains lysogenic for 80α phage and containing SaPI1 were grown to early exponential phase (OD_540_ ∼ 0.15) at 37°C and 120 rpm. Cultures were then induced by the addition of MC (2 μg/ml) and incubated for 4–5 h at 30°C followed by overnight incubation at room temperature before filtering the lysate with a 0.2 μm syringe filter. For SaPI titre determination, *S. aureus* RN4220 strain was grown overnight at 37°C and 120 rpm. The culture OD was adjusted to OD_540_ ∼1.4 with TSB and supplemented with 4.4 mM CaCl_2_. 100 μl of the appropriate lysate dilution were added to 1 ml of this cell suspension and incubated for 30 min at 37°C. Three ml of transduction top agar (TTA, 30 g/l TSB, 7.5 g/l agar) were added to the transduction and the mix poured onto a TSA plate containing the appropriate antibiotic. Plates were incubated for 16–24 h at 37°C prior to determination of transducing units.

### Size-exclusion chromatography small angle X-ray scattering (SEC-SAXS)

SEC-SAXS was done on beamline B21 of the Diamond Light Source synchrotron (Didcot, UK). Data were recorded at 12.4 keV, at a sample-detector distance of 4.014 m using a Pilatus 2 M detector (Dectris, Switzerland). 50 μl of protein samples at concentrations of 10.0 mg/ml (Stl^SaPI1^) and 6.5 mg/ml (Stl^SaPI1^-Sri) were loaded onto a Superdex 200 Increase 3.2 size exclusion chromatography column in 10 mM Tris pH 8, 1% (w/v) sucrose at 0.075 ml/min using an Agilent 1200 HPLC system. The column outlet was fed into the experimental cell, and 620 × 3.0 s frames of SAXS data were recorded. Data were processed with ScÅtter IV (http://www.bioisis.net) as follows. The integral of ratio to background signal along with the estimated radius of gyration (*R*_g_) for each frame was plotted. Frames within regions of low signal and low *R*_g_ recorded prior to protein elution were selected as buffer and subtracted from frames within regions of higher signal and constant *R*_g_. Subsequent analysis was performed using the ATSAS 3.0 suite of programs (20). The radius of gyration *R*_g_ was obtained from the Guinier approximation following standard procedures. The pairwise distance distribution function *p*(*r*) was computed using the indirect Fourier transformation method implemented in GNOM (21). From the *p*(*r*) function, an alternative estimate of *R*_g_ and the maximum particle dimension *D*_max_ were obtained. Molecular weights were estimated by Bayesian inference (22) in Primus (20). Ensemble optimization modelling was undertaken with EOM (23). 10 000 models were generated in which the C-terminal domains (CTD, residues 101–247) were kept in the conformation observed by X-ray crystallography but the DBDs (residues 1–89) allowed to adopt positions consistent with their connection to the CTD via a native-like flexible linker (residues 90–100).

### Identification of Stl^SaPI1^ homologs

The search for Stl^SaPI1^ homologs was done using the Blast server. The structural models for the Stl^SaPI1^ homologs were predicted using the AlphaFold server (24) with the option ‘template mode: none’ and the structural alignments were performed with PROMALS3D (25). All homolog model representations were generated with UCSF Chimera (26).

### Quantification and statistical analysis

All statistical analyses were performed as indicated in the figure legends using GraphPad Prism 6.01 software.

## RESULTS

### SaPI1 Stl is a tetramer

To understand the molecular basis of SaPI1 Stl (Stl^SaPI1^) repression, we initiated these studies to solve its atomic structure, either alone or bound to its cognate operator. Although this was not possible, during its purification the chromatographic assays revealed that Stl^SaPI1^ is a tetramer in solution. The Stl^SaPI1^ monomer has 244 residues with a predicted molecular weight (MW) of 29.9 kDa (without the N-terminal His^6^-tag), but size-exclusion chromatography multi-angle light scattering (SEC-MALS) eluted Stl^SaPI1^ in a single peak with a calculated MW of 117.4 kDa, which corresponds to four molecules of Stl^SaPI1^ (Supplementary Figure S1). This result was interesting because most MGE repressors studied to date form dimers. It raised interesting questions about how Stl^SaPI1^ performs its function and how the SaPI1 inducer, phage 80α Sri protein, promotes SaPI1 induction. Our initial hypothesis was that Sri disrupts the Stl^SaPI1^ oligomeric state, as was observed for Stl^SaPIbov1^. However, SEC-MALS analysis of Sri in complex with Stl^SaPI1^ demonstrated that the tetrameric form was not affected by the presence of the inducer (Supplementary Figure S1). Co-expression of Sri and Stl^SaPI1^ formed a complex that eluted later than Stl^SaPI1^ alone and showed a smaller hydrodynamic radius, despite a molecular weight of 128 kDa corresponding to one Stl^SaPI1^ tetramer plus 1 or 2 Sri molecules bound to the tetramer (Supplementary Figure S1). This result suggested that the Stl^SaPI1^ tetramer alone has a structure that is more extended than when it is in a Sri-Stl^SaPI1^ complex, indicating that Sri has the effect of compacting the Stl^SaPI1^ tetramer.

**Figure 1.**
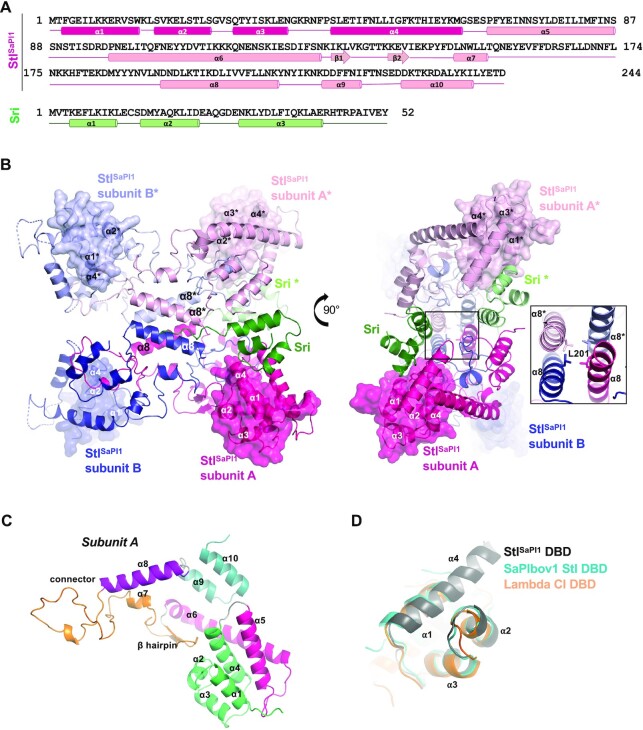
Structure of the Stl^SaPI1^–Sri complex. (**A**) Representation of the secondary structure of Stl^SaPI1^ (in pink) and 80⍺ Sri (in green) proteins. The four first α helices of the Stl^SaPI1^ DBDs are coloured in dark pink. (**B**) Two different views of the 80⍺ Sri-Stl^SaPI1^ complex structure. The molecules in the symmetric unit of the crystal are coloured in dark pink and blue for Stl^SaPI1^ and green for Sri, while the asymmetric molecules are coloured in light colours. The Stl^SaPI1^ DBD surfaces are highlighted in semi-transparent shading. The right part of the figure shows the Stl^SaPI1^ L201 residue located in the helix α8, its sidechain is represented as stick. (**C**) Structure of the Stl^SaPI1^ subunit A obtained from the Sri-Stl^SaPI1^ complex. The DBD is coloured in green, the helices α5 and α6 are in pink, and the central part of the molecule (β hairpin, α7 and α7–α8 connection) is in orange. The helix α8 is in purple and the C-terminal part (helices ⍺9 and ⍺10) is in blue. (**D**) Superimposition of the Stl^SaPI1^ DBD in black, the SaPIbov1 Stl DBD (PDB: 6H49) in blue, and the CI lambda phage repressor DBD (PDB: 1LMB) in orange. The four alpha helices are marked.

The SEC-MALS results were confirmed by the X-ray crystallographic structure of the Sri-Stl^SaPI1^ complex (Figure 1), which was determined to 2.9 Å resolution by single-wavelength anomalous dispersion (SAD) using the diffraction of a selenomethionine-substituted derivative crystal (Table 1). The asymmetric unit of the crystal contained two Stl^SaPI1^ monomers (subunits A and B) in a dimeric organization around a non-crystallographic two-fold axis, with one Sri molecule binding to the Stl^SaPI1^ subunit A (Figure 1B). The electron density observed in the model suggested that a second Sri molecule binds to the corresponding position of Stl^SaPI1^ subunit B. However, in concordance with our SEC-MALS results, this additional Sri molecule showed an extremely low occupancy that allowed us to only trace the main chain of fewer than 36% of its residues. In correlation with the low occupancy of the second Sri molecule, two regions of Stl^SaPI1^ subunit B (residues 32–47 and 83–97), which mediate interactions with the inducer in subunit A and connect the DBD and the central part of the protein, present high flexibility that also prevents their tracing. Similarly, we were unable to trace the six N-terminal Stl^SaPI1^ residues which are also placed in this area on subunit A, supporting the idea that the presence of Sri stabilizes the Stl^SaPI1^ N-terminal region. In agreement with our structure and the SEC-MALS experiments, assembly analysis with the PDBePISA server (27) indicated that the Stl^SaPI1^ dimer forms a stable tetramer (a dimer of dimers) exploiting the crystallographic two-fold axis. Therefore, the biological assembly of Sri-Stl^SaPI1^ is a box-shaped hetero-octamer of dimension 95 × 95 × 45 Å, containing two Stl^SaPI1^ homodimers (subunits A–B and A*–B*) and four Sri molecules, two of which were tightly bound (subunits A and A*) and two others with partial occupation (subunits B and B*). In fact, we noticed that some Sri protein eluted alone in a later single peak in our SEC assays. Since Sri can be purified only in a complex with Stl^SaPI1^, the fact that we observed a Sri peak in the chromatographic purification (Supplementary Figure S2) suggested that some Sri molecules were released from the complex because they were weakly bound to Stl^SaPI1^ and/or because the Sri-Stl^SaPI1^ complex has a large dissociation rate constant.

Each Stl^SaPI1^ subunit presents an N-terminal DNA binding domain (DBD) with a conserved HTH-XRE motif formed by 4 α helices (helix α1-α4; residues 1–67) (Figure 1C). This domain is similar in structure to the one in Stl^SaPIbov1^ (PDB ID: 6H49) (9) and in many phage repressors, including phage λ CI (PDB ID: 1LMB) (28), showing the superimposition of Stl^SaPI1^ DBD with the equivalent Cα atoms of these repressors with a higher root-mean-square deviation (RMSD) of 1.40 and 2.3 Å, respectively (Figure 1D). Conversely, no similar structures were found in the PDB for the rest of the Stl^SaPI1^ repressor. A search for 3D homologs by comparison servers such as DALI or PDBefold (29) (30), using as prey the structure formed by Stl^SaPI1^ residues 68–244, did not identify any structural homologs to Stl^SaPI1^. This unique architecture is composed of two long α helices (α5 and α6) with a long loop between them that connects the DBDs to the central part of the protein. This central part is formed by two antiparallel strands (β1-β2) in a β hairpin and a short α helix (α7), followed by an extended region without secondary structure (residues 157–191) that projects from one subunit onto the other in the dimer, then returns to the same subunit via a long helix (α8). Finally, two shorter helices (α9 and α10) form the C-terminal part of the protein (Figure 1C). Thus, each subunit is highly elongated with a distance of more than 80 Å between the DBDs and the unstructured region of the protein.

Stl^SaPI1^ self-associates in a unique conformation that has not been previously observed in other repressors, with more than 40% of its residues interacting to form a huge dimerization surface of ∼9230 Å^2^ per subunit (see Supplementary Table S7 for a detailed description of the interaction between the two subunits). In the dimer, 4 helices (α4, α5, α9 and α10) plus the β harpin of one subunit create a cavity where connecting helices α7* and α8* (residues 154–183) from the other subunit are located, embracing one monomer with the other (Supplementary Figure S3). Although a large number of contacts maintain Stl^SaPI1^ dimers, the α7–α8 connector assembles the most important interactions for dimer formation (Supplementary Table S7). A comparison of Stl^SaPI1^ subunits A and B showed an overall RMSD of 1.5 Å (superimposition of 207 Cα atoms). The differences between both subunits were not uniform along the molecule, being higher towards the N-terminus (RMSD > 2.5 Å) where the HTH domains are located. By contrast, the differences were smaller in the main body of the molecule (RMSD < 1 Å), which is used for oligomerization. These results suggest a high plasticity for the tetramer, which is reflected in the mobility of the DBDs.

Further analysis of the tetrameric state of Stl^SaPI1^ revealed that it presents a reduced oligomerization interface that buries only ∼2150 Å^2^ of the tetramer surface (∼540 Å^2^ per subunit), supporting a dimer-of-dimers organization for the Stl^SaPI1^ tetramer. The tetramerization surface is generated mainly by the mutual interaction of helices α8 (residues 194–209) from each subunit, forming an anti-parallel four-helix bundle in the tetramer (Figure 1B). These interactions were mainly hydrophobic and provided by the sidechains of residues N193, D194, T197, D200, L201, V204, F205, N208 and K209 that face the centre of the tetramer (Supplementary Table S8).

### Stl^SaPI1^ tetramerization is necessary for SaPI1 repression

To gain more insight into the biology of the Stl^SaPI1^ repressor, we analysed whether the tetramer was required for SaPI1 repression. Our previous structural analysis suggested the importance of residue L201 for tetramerization since their mutual interaction projecting from the middle of α8 nucleates the hydrophobic core (Figure 1B, Supplementary Table S8). In support of this, a Stl^SaPI1^ repressor carrying an L201E mutation (Stl^SaPI1 L201E^), which introduces a charged residue into this hydrophobic environment, formed dimers in solution (Supplementary Figure S1) and confirmed the role of this helix in Stl^SaPI1^ tetramerization. Furthermore, we solved the structure of the Sri-Stl^SaPI1 L201E^ complex, confirming both the dimerization state of the L201E mutant and its ability to interact with Sri, as our previous co-expression and SEC-MALS experiments suggested (Supplementary Figure S1).

**Figure 2.**
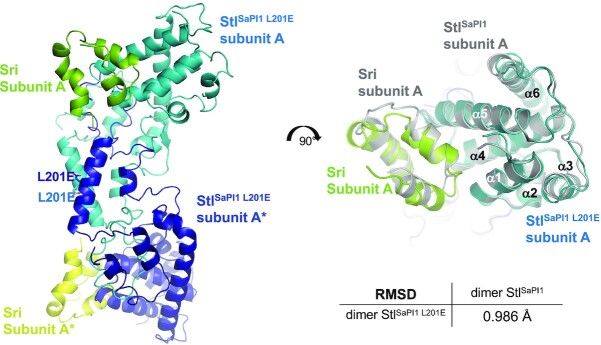
Structure of Sri-Stl^SaPI1 L201E^ complex. Structure of the Sri-Stl^SaPI1 L201E^ dimer complex obtained by X-ray crystallography. The Stl^SaPI1 L201E^ dimer is colored in blue, while the Sri molecules are coloured in green and yellow. The sidechain of the mutated E201 residue is represented as stick. The right part of the figure represents an apical view of the Sri-Stl^SaPI1 L201E^ structure superimposed with the wt Sri-Stl^SaPI1^ dimer seen in the asymmetric unit of the crystal (subunits A and B, in grey). The RMSD between the wt and the L201E Stl^SaPI1^ dimers is represented.

The Sri-Stl^SaPI1 L201E^ structure was determined at 2.97 Å resolution by molecular replacement using the wt Sri-Stl^SaPI1^ monomer structure that was previously determined as a model. The asymmetric unit of the crystal showed a monomer of Stl^SaPI1 L201E^ bound to a molecule of Sri (Figure 2). This complex formed a dimer with the symmetric molecules with identical organisation to that observed for the dimer formed by subunits A and B in the crystal asymmetric unit of the wt Sri-Stl^SaPI1^ complex (RMSD of 1.2 Å for the superimposition of 489 Cα atoms of wt dimer with mutant crystallographic dimer), confirming the A–B subunit organization for the dimeric Stl^SaPI1^ (Figure 2). Stl^SaPI1^ plasticity was also observed when comparing the subunits A and B of the wt Stl^SaPI1^ with those of the Stl^SaPI1 L201E^ mutant (RMSDs of 0.9 and 1.6 Å for the superimposition of 243 and 208 Cα atoms of mutant Stl^SaPI1^ with the wt subunits A and B, respectively).

The Sri molecule binds to Stl^SaPI1 L201E^ in a disposition identical to that previously observed in the wt Sri-Stl^SaPI1^ complex, indicating that the L201E mutation did not affect Stl^SaPI1^ binding to Sri (Figure 2). However, although the Sri fold in both wt and mutant Stl^SaPI1^ complexes is identical (RMSD of 1.5 Å for superimposition of 42 Cα atoms), the 7 C-terminal residues were not visible in the complex with the Stl^SaPI1 L201E^ mutant. In the complex with wt Stl^SaPI1^ protein, this portion of Sri was projected into the tetramerization region where it made contact with helix α8. Loss of tetramer organization by the mutation could be detrimental to the stabilization of this region and consequently for complex formation (see below). Corroborating this, the Sri-Stl^SaPI1 L201E^ complex was unstable during the purification process and we observed Sri released from the complex during gel filtration chromatography (Supplementary Figure S2).

**Figure 3.**
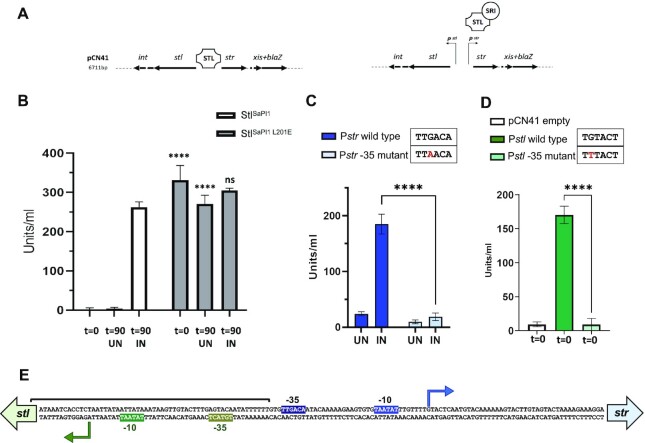
The Stl^SaPI1^ tetramer is required for SaPI1 repression. (**A**) Schematic representation of the pCN41 derivative reporter plasmid used to analyse Stl^SaPI1^ repression. In the absence of prophage induction, the expression of SaPI1 and *blaZ* genes is repressed by Stl^SaPI1^. Induction of the 80α prophage results in expression of the SaPI1 inducer Sri, which promotes the expression of the SaPI1 genes, including the *blaZ* reporter. (**B**) Lysogenic strains for phage 80α, carrying pCN41 derivative plasmids expressing either the wt Stl^SaPI1^ or the mutant Stl^SaPI1^ L201E, were MC-induced (IN) or not (UN), and the expression of the *blaZ* reporter analysed at time zero (*t* = 0) or 90 min (*t* = 90) after MC induction. The means and standard deviation from three independent experiments are represented. A two-way ANOVA comparation was performed to compare the different samples mean between Stl^SaPI1^ and Stl^SaPI1 L201E^ (****P < 0.0001; ns, *P* = 0.1234). (**C**) Characterization of the *str* promoter. The strains lysogenic for phage 80α, carrying pCN41 derivative plasmid containing either the wt *str* promoter (P*str*), or one carrying a point mutation in the putative –35 site of the P*str* (mutant P*str)*, were MC-induced (IN) or not (UN) and the expression of the *blaZ* reporter was analysed 90 min after prophage induction. The means and standard deviation from three independent experiments are represented. A two-way ANOVA comparation was performed to compare the IN wt P*str* mean with the IN P*str* mutant mean (****, *P* < 0.0001). (**D**) Characterization of the *stl* promoter. RN4220 strains carrying pCN41 derivative plasmids containing either the wt *stl* promoter (P*stl*) or one carrying a point mutation in the putative P*stl* –35 region (P*stl* mutant), were analyzed at zero min. The means and standard deviation from three independent experiments are represented. A one-way ANOVA comparation was performed to compare the wt P*str* mean with the P*str* mutant mean (****P < 0.0001). (**E**) DNA sequence of the SaPI1 *stl-str* intergenic region. The transcription start sites are represented with arrows and the –10 and –35 sequences are highlighted in blue for the P*str*, or in green for the P*stl*. The *stl* promotor region that was cloned into the pCN41 plasmid for the characterization of the P*stl* is marked with a bracket.

To test whether the dimeric Stl^SaPI1 L201E^ was able to repress SaPI1, we generated a plasmid in which a β-lactamase reporter gene was fused to *xis*, downstream of *str* and the Stl-repressed *str* promoter. This plasmid also encodes the *stl* in the opposed direction (see scheme in Figure 3A). As a control, we generated a derivative plasmid expressing Stl^SaPI1 L201E^. These plasmids were introduced into strain RN4220 lysogenic for the SaPI1 helper phage 80α (5). These strains were then treated mitomycin C (MC) for prophage induction, samples were taken at time zero and after 90 minutes, and the expression of β-lactamase was quantified. In accordance with previous studies (4,5), Stl^SaPI1^ blocked β-lactamase expression in the absence of prophage induction (Figure 3B). Prophage activation expressed the Sri inducer, which derepressed the system promoting the expression of β-lactamase (Figure 3B). In contrast, the plasmid expressing the dimeric Stl^SaPI1 L201E^ repressor showed extremely high reporter expression even in the absence of prophage induction (Figure 3B), confirming its inability to cause repression. Note that we tried to generate a SaPI1 derivative island encoding the Stl^SaPI1^ L201E mutation, but this was not possible since mutations that negatively affect Stl function are not stable owing to uncontrolled replication of the SaPI (3). In summary, these results demonstrate that the tetrameric structure is required for Stl^SaPI1^ repression.

### Characterization of the *stl* and *str* promoter regions

Next, we asked why the tetrameric Stl^SaPI1^ is required for SaPI1 repression. To do this, we performed 5′-RACE to identify the *stl* and *str* transcription start sites. We also localized the putative RNA polymerase binding sites for both promoters (Figure 3E). While the *str* promoter showed canonical –10 and –35 sequences, the –35 site of the *stl* promoter was degenerate (Figure 3E), suggesting that the *str* promoter was likely stronger than the *stl* promoter (31).

To validate the localization of the putative *str* promoter, we made use of the reporter plasmid in which the *bla*Z reporter gene was fused to *xis* (see scheme in Figure 3A), but now with a single nucleotide mutation in the –35 site of the *str* gene. This plasmid was introduced into the strain lysogenic for 80α and the expression of β-lactamase was measured. We found that the single nucleotide mutation eliminated transcription of the *str* promoter after prophage induction (Figure 3C).

To characterize the *stl* promoter, we made a transcriptional fusion of the *stl* promoter to the *bla*Z reporter gene (see Figure 3E). We also generated a derivative with a single point mutation in the –35 site of the *stl* promoter. These plasmids were then introduced into the non-lysogenic RN4220 strain and the expression of reporter was measured. While the wt plasmid showed high β-lactamase expression, the plasmid carrying the point mutation did not (Figure 3D). Taken together, point mutations in the –35 sites of the *str* and *stl* promoters completely abolished their transcription, confirming the identity of both promoters.

### The SaPI1 *stl-str* intergenic region contains 8 Stl^SaPI1^ binding sites

**Figure 4.**
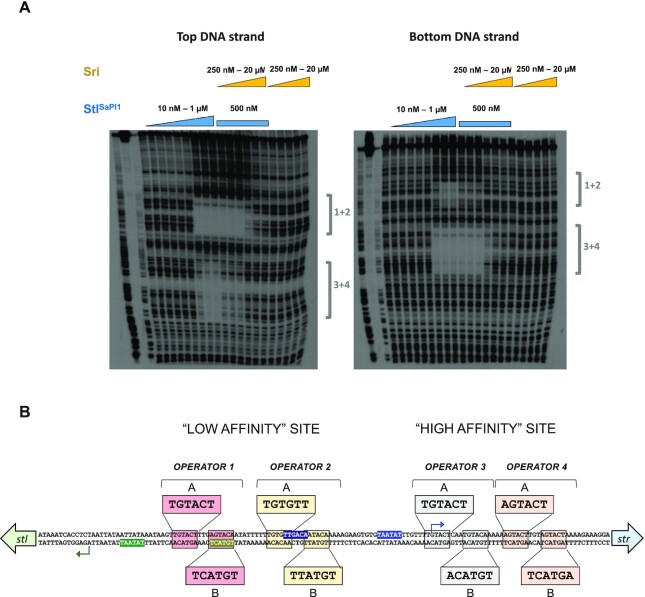
Identification of the Stl^SaPI1^ binding sites in the SaPI1 *stl-str* intergenic region. (**A**) Foot-printing experiments carried out with the SaPI1 *stl-str* intergenic region and the Stl^SaPI1^ protein, alone or in the presence of Sri. Protein concentrations used in the experiment are represented in the figure. The protected regions associated with operators 1 and 2 (1 + 2) or 3 and 4 (3 + 4) are shown. Regions 1 + 2 and 3 + 4 correspond to regions with low and high affinity for Stl^SaPI1^, respectively. (**B**) Schematic representation of the four operator sites for Stl^SaPI1^. Both palindromic boxes of each operator are highlighted as A and B. The P*str* and P*stl* transcription start sites are represented by blue or green arrows, respectively. The –10 and –35 sequences from both promotors are also highlighted in blue and green, respectively.

We next focused on the SaPI operators to determine if their organization reflected the four DBDs in a Stl^SaPI1^ tetramer. Foot-printing experiments using Stl^SaPI1^ with the SaPI1 *stl-str* intergenic region revealed two protected regions, separated by 24 bp, in the top and bottom strands (Figure 4A). We used this intergenic region because previous studies have shown that SaPI Stl proteins bind to it (5,9), and our reporter assays showed that it is regulated by Stl. Detailed analysis of the protected regions identified eight putative Stl^SaPI1^ binding sites organized as four distinct operators (1–4) (Figure 4B). Operators 3 and 4 appear to represent a higher affinity site because they were fully protected at lower concentrations of Stl^SaPI1^ and required a higher concentration of Sri to lose protection compared with the region containing operators 1 and 2. Each of the putative operators shows almost perfect palindromic organization, containing two inverted 6 bp repeats with the consensus sequence TGTACT (called boxes A and B) separated by 3 bp (Figure 4B). Importantly, operators 1 and 2 overlap with the -35 sites of the *stl* and *str* promoters, suggesting that Stl^SaPI1^ represses by blocking the binding of RNA polymerase to the *str* and *stl* promoter regions.

**Figure 5.**
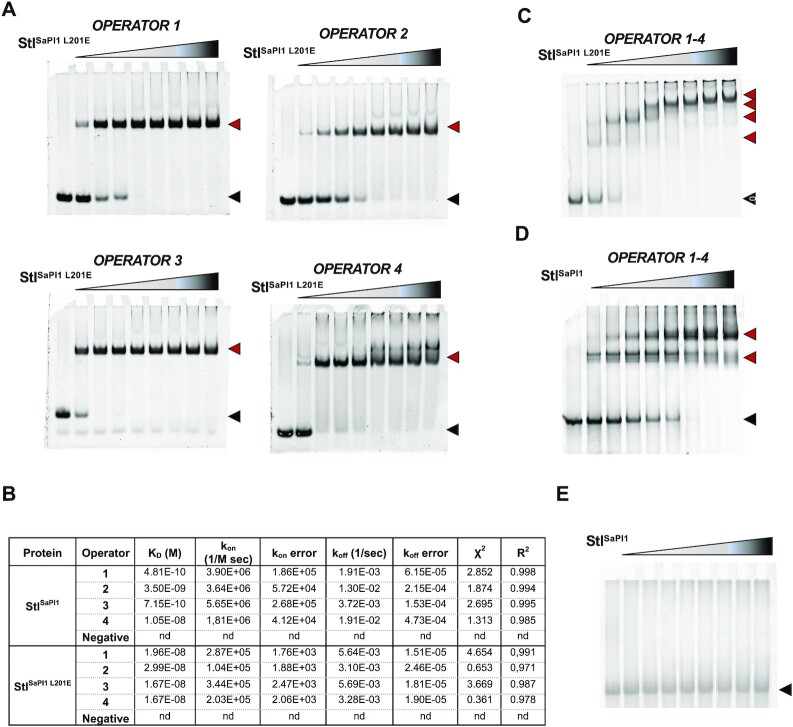
Characterization of the Stl^SaPI1^ binding sites present in the *stl-str* intergenic region. (**A**) EMSAs performed using the Stl^SaPI1 L201E^ mutant protein (0.5, 1, 1.5, 2, 2.5, 3, 3.5 and 4 μM per well), and DNA probes (1 μM) containing only one operator site per probe. The free probe band is marked with a black arrow, while the Stl-DNA complex bands are highlighted with a red arrow. (**B**) Biolayer interferometry assays with both the Stl^SaPI1^ and the Stl^SaPI1 L201E^ proteins and each of the probes containing one of the four individual operators in the *stl-str* intergenic region. A negative probe, operator 3 with the Stl binding sites mutated to Adenines, was used as nonspecific DNA binding control. ‘*K*_D_’ affinity constant, ’*k*_on_’ association constant, ‘*k*_off_’ dissociation constant, ‘nd’ non signal detected. (**C**) As for (A) but using Stl^SaPI1 L201E^ mutant protein (1, 2, 3, 4, 5, 6, 7 and 8 μM per well) and a DNA probe (1 μM) containing the full *stl-str* intergenic region with the four operators. (**D**) As for (C) but using the wt Stl^SaPI1^ protein. (**E**) As for (D) but using a negative probe with the four operators with the consensus regions mutated to Adenines.

Since classical repressors containing HTH-XRE domains are usually dimers that bind to palindromic operators, the existence of four operators suggested that two Stl^SaPI1^ tetramers bind to this region (corresponding to four classical dimers). To test this, we first confirmed that the 4 identified operators are recognized and bound by the Stl^SaPI1^ DBDs. To simplify the interpretation of our results, we initially performed electrophoretic mobility shift assays (EMSA) using the Stl^SaPI1 L201E^ mutant since the altered residue does not affect the conformation of the DBD domain or its ability to bind operators. We hypothesized that each of the DBDs in the Stl^SaPI1 L201E^ mutant would bind as a dimer to each of the four operators, independently. To analyse the binding of Stl^SaPI1 L201E^, we generated a set of DNA probes that each contained one of the operators. As shown in Figure 5A, Stl^SaPI1 L201E^ bound all 4 DNA probes, showing the highest affinity for operators 3 and 4 and corroborating the foot-printing results.

Next, protein–DNA binding kinetics assays were performed by biolayer interferometry to obtain the affinity constant (*K*_D_) of both the wt Stl^SaPI1^ and the L201E mutant protein with the four individual operators previously described. A DNA probe of operator 3, with the binding boxes mutated to A, was also used as a negative control. The results showed that Stl^SaPI1^ bound to each of the operators with *K*_D_ values ranging from 0.7 to 10 nM (Figure 5B), while Stl^SaPI1L201E^ showed similar *K*_D_ values of 17–30 nM. Of note, the constants for operators 1, 2 and 3 were 1 to 2 orders of magnitude lower than those observed for the tetrameric wt Stl^SaPI1^ repressor (Figure 5B). No binding was observed with the mutated operator 3, confirming DNA recognition specificity. While the binding kinetics for the Stl^SaPI1 L201E^ mutant fit a 1:1 model (operator:dimer), we observed more complex sensograms with wt Stl^SaPI1^ which better corresponded to a mass transport model (Supplementary Figure S4). These observations also explain the differences in the *K*_D_ values observed between the wt and the mutant Stl^SaPI1^, since with the wt protein we visualized an avidity effect owing to the tetramer that simultaneously bound two independent and immobilized operators (one per dimer). These results also indicated that Stl^SaPI1^ and Stl^SaPI1 L201E^ bound operators through the dimer formed by subunits A and B.

### Two Stl tetramers bind to the *stl-str* intergenic region

Having demonstrated the existence of four operators in the *stl-str* intergenic region, we then analysed the interaction of the wt Stl^SaPI1^ and the Stl^SaPI1 L201E^ mutant with a DNA probe containing all four operators. Here, four different protein–DNA species were observed in the presence of the Stl^SaPI1 L201E^ mutant protein (Figure 5C); interestingly, only two different protein–DNA species were obtained with the wt Stl^SaPI1^ (Figure 5D). As a control, a probe with all of the consensus sequences mutated to adenine was used to confirm DNA-binding specificity of the different proteins (Figure 5E). SEC-MALS characterization of the sample corresponding to the DNA probe in the presence of an excess (4-fold molar ratio) of wt Stl^SaPI1^ showed two peaks. The first peak had a calculated MW in close agreement to that of a complex of two tetramers with one DNA probe, while the second peak had a MW almost identical to that of a Stl^SaPI1^ tetramer (Supplementary Figure S5) that was likely attributable to the excess protein. Both the EMSA and SEC-MALS results supported the hypothesis that Stl^SaPI1^ tetramers are bivalent and bind two DNA operators. Therefore, two tetramers would be enough to occupy the four operators in *stl-str* intergenic region.

**Figure 6.**
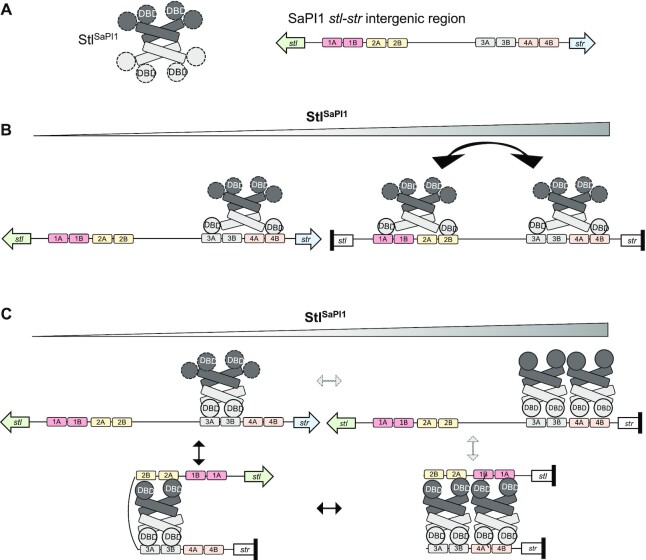
Proposed models for Stl^SaPI1^ repression. (**A**) Representation of the Stl^SaPI1^ tetramer and the SaPI1 *stl-str* intergenic region. The two dimers that from the Stl^SaPI1^ tetramer are dark and light grey and the mobility of the DBDs is represented by dashed lines. The 4 operators are represented with different colors and the inverted repeats are named A and B. (**B**) Model 1. When Stl^SaPI1^ concentration is low, one tetramer binds the high-affinity operators 3 and 4, with the DBDs from the dimer binding to 3A and 4B to repress *str* expression. When Stl^SaPI1^ concentration increases, a second tetramer binds to 1A and 2B to repress *stl* expression. Since tetramer formation is required for SaPI1 repression, the two tetramers interact somehow to stabilize the complex. (**C**) Model 2. At low Stl^SaPI1^ concentration, one dimer of the Stl^SaPI1^ tetramer binds to both repeats (A and B) from operator 3, while the other binds to the repeats present in operator 2, creating a torsion in the DNA which favors *str* repression. When Stl^SaPI1^ concentration increases, a second tetramer binds in a similar manner to operators 1 and 4, stabilizing the complex and increasing repression of the system, represented in black arrows. In grey arrows an alternative model is represented with a sequential entry of the tetramers, two tetramers could bind simultaneously to operator 3 and 4 by one of their dimeric parts, inducing DNA torsion by the binding of their second dimeric part to operators 2 and 1, respectively.

Our previous results showed that Stl^SaPI1 L201E^ was unable to repress SaPI1, even though it binds to the 4 Stl boxes with high affinity. This result implied that the Stl^SaPI1^-DNA interaction was more complex than just DNA binding. The existence of 4 operators with different affinities for Stl^SaPI1^, together with the requirement for 2 Stl^SaPI1^ tetramers with high flexibility in their DBD domains, prompted us to propose two different models for SaPI1 repression (currently under investigation) (Figure 6). In the first model, which is supported by the foot-printing experiments, one tetramer binds with high affinity to operator sites 3 and 4, while the other tetramer binds to sites 1 and 2. In order to bind both sites at the same time, Stl^SaPI1^ must recognize an inverted palindrome of each operator that would generate two canonical operators (3A-4B and 1A-2B). In this case, the binding sites are separated by 22–27 bp instead of 3 bp spacer (Figure 6B). The length of this spacer would imply that the DNA-binding helices must be more than 60 Å apart, which should not be a problem for Stl^SaPI1^ since the Sri-Stl^SaPI1^ structure shows that the α3 helices are separated by ∼61 Å in the dimer. Although this type of separation between palindromes is unusual, it has recently been observed that the transcriptional activator AimR from *B. subtillis* phage SPbeta recognizes an operator with similar organization, where highly flexible DBDs more than 75 Å apart bridge a 25 bp spacer (32,33). A protein-protein interaction between both tetramers could create a bigger protein–DNA complex to stabilize Stl repression, which could explain why Stl^SaPI1^ tetramers are required for SaPI1 repression. Another possibility is that one dimeric part of the first tetramer initially binds with high affinity to operator 3, and then the binding of the second dimeric part of this tetramer to operator 2 induces a DNA torsion that is facilitated by the high A/T content of the inter-operator spacer (Supplementary Figure S6). Next, a second tetramer stabilizes the protein–DNA complex via bivalent binding to operators 1 and 4 (Figure 6C, Supplementary Figure S6). An alternative to the sequential entry of tetramers in this model, two tetramers could bind simultaneously to operators 3 and 4 by one of their dimeric parts, inducing DNA torsion by the binding of their second dimeric parts to operators 2 and 1, respectively. Such multiple interactions would be possible because of the high flexibility observed in the Stl^SaPI1^ DBDs (see below), and because of the ability of these domains to bind, bend and twist DNA. We propose that either of these models could stabilize the complex, preventing RNA polymerase from binding to the *stl* and *str* intergenic region - a process that is also supported by the observation that Stl^SaPI1^ binds to the –35 sites of the *stl* and *str* promoter (located in operators 1 and 2, respectively; Figure 4B). Importantly, in the case of the *stl* promoter, this repression would reduce Stl^SaPI1^ formation. Therefore, both models propose that the second tetramer controls the amount of Stl^SaPI1^ that will be produced, which is crucial for the control of the system. When Stl^SaPI1^ is in excess, binding of the second tetramer to operator 1 will reduce *stl* expression; when Stl^SaPI1^ decreases, the absence of the second tetramer will allow *stl* expression. Although we have not yet confirmed either model, binding of Stl^SaPI1 L201E^ to individual operators and the EMSA results observed for wt and mutant repressors support the latter model, while the foot-printing data are more consistent with the former. Further studies are required to resolve this mechanism.

### Molecular basis of SaPI1 de-repression by Sri

Our results demonstrate that in each operator, a dimer with two Stl^SaPI1^ DBDs binds to an operator with two palindromic sequences separated by 3 bp. However, in the X-ray crystallography structure of the Sri-Stl^SaPI1^ complex, the distance observed between the two DBDs (>60 Å) was greater than that required to bind to the Stl boxes in the operators (30 Å, Supplementary Figure S7). Sri is a 6.2 kDa protein (52 residues) composed of a 3-helix bundle (α1–α3) followed by an extended non-structured C-terminal tail (residues 43–51) (Figure 1). A DALI search showed that Sri is structurally similar (1.28 Å RMSD over 41 Cα superimposed) to the phage 77 ORF104, whose structure was previously solved in complex with DnaI, its cellular partner (34) (PDB ID 5HE9). When complexed with Stl^SaPI1^, Sri is inserted in the Stl^SaPI1^ tetramer, interacting with 3 of the 4 Stl^SaPI1^ subunits and burying ∼1550 Å^2^ of its surface, which corresponds to ∼33% of the Sri molecular surface. Each Sri molecule mainly interacts with the Stl^SaPI1^ DBD of one subunit by using helices α2 and α3 to contact Stl^SaPI1^ helices α1, α4 and α5 (Supplementary Table S9). In addition, the Sri α3 helix interacts with the C-terminal α10 helix of the symmetrically related subunit (A*) in the Stl^SaPI1^ tetramer; and its extended C-terminal tail is positioned over two α8 helices (subunits A* and B), which nucleates Stl^SaPI1^ tetramerization by interactions with subunit B (Supplementary Table S9). By performing this network of interactions, Sri fixes the Stl^SaPI1^ DBDs to the main body of the Stl^SaPI1^ tetramer, restricting its conformational freedom and compacting the Stl^SaPI1^ tetramer structure, as our SEC-MALS experiments corroborate (Supplementary Figure S1). Low occupancy of Sri in the second binding site of the Stl^SaPI1^ dimer correlates with the high flexibility of the DBD and supports Sri function. Therefore, we hypothesize that Sri de-represses SaPI1 by fixing the Stl^SaPI1^ DBDs (Figure 1, Supplementary Table S9) in a conformation that is not compatible with the binding of the Sri-Stl^SaPI1^ complex to the Stl^SaPI1^ operators in the *stl*-*str* intergenic region (Supplementary Figure S7).

**Figure 7.**
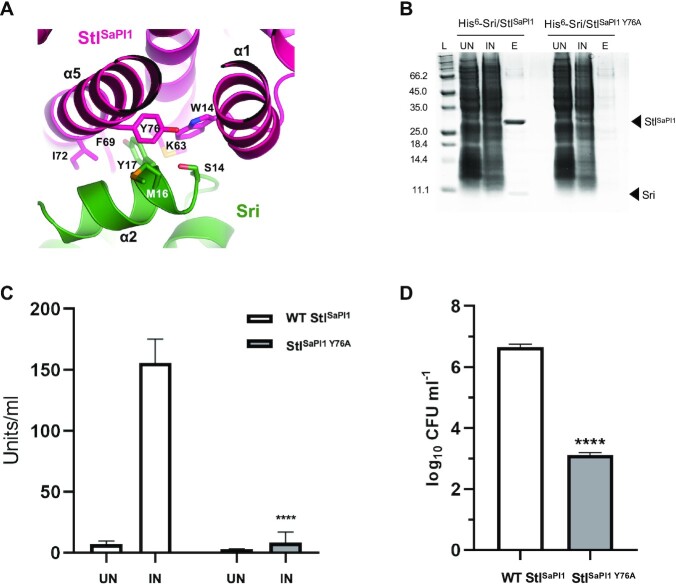
Characterization of the Stl^SaPI1^-Sri interaction. (**A**) Details of the Stl^SaPI1^ (in pink) and Sri (in green) interaction. The Stl^SaPI1^ Y76, S13, M16 and K20 Sri residue sidechains are represented as sticks. (**B**) SDS-PAGE gel after pull-down experiments in which the His^6^ tagged Sri protein was co-expressed either with the wt Stl^SaPI1^ or the Stl^SaPI1 Y76A^ proteins. Uninduced (UN), induced (IN) and eluted (**E**) from the Ni^2+^ column. Note the absence of the prey band due to the loss of solubility of Sri in the absence of Stl^SaPI1^. (**C**) Strains lysogenic for phage 80α, carrying pCN41 derivative plasmids expressing either wt Stl^SaPI1^ or Stl^SaPI1 Y76A^, were MC-induced (IN) or not induced (UN) and expression of the *blaZ* reporter analysed 90 min after prophage induction. The means and standard deviation from three independent experiments are represented. A t-test comparation was performed to compare IN Stl^SaPI1 Y76A^ mean with IN Stl^SaPI1^ mean (****P < 0.0001). (**D**) Lysogenic strains for phage 80α, carrying wt SaPI1 *tst*::*tet*M or a derivative SaPI1 *tst*::*tet*M carrying the Stl^SaPI1^ Y76A mutation, were MC-induced (IN) or not induced (UN), and the transfer of the island quantified. The means and standard deviation from three independent experiments are represented. A t-test comparation was performed to compare each the Stl^SaPI1 Y76A^ mean with the Stl^SaPI1^ mean (****P < 0.0001).

To test this hypothesis, we first validated our structural data for the Stl^SaPI1^-Sri complex. To do this, we mutated the Stl^SaPI1^ residue Y76 to Alanine (Stl^SaPI1 Y76A^), which we deduced was important for the stabilization of the Stl^SaPI1^-Sri complex by projecting its side chain into a hydrophobic pocket generated by Stl^SaPI1^ residues W14, M63, F69, I72 and Y76 (Figure 7A, Supplementary Table S8). Pull-down assays confirmed that the Stl^SaPI1 Y76A^ repressor was unable to bind to Sri (Figure 7B). To show that this mutation affected only the interaction with Sri, but not the ability of Stl^SaPI1 Y76A^ to repress the island, we again used the β-lactamase reporter plasmid (see scheme in Figure 3A) with either the wt or the Stl^SaPI1 Y76A^ mutant repressor. These plasmids were introduced into the 80α lysogen and expression from the Stl-repressed *str* promoter was measured after induction of the 80α prophage. Compared with that observed for wt Stl^SaPI1^, no significant activity was observed in the plasmid expressing Stl^SaPI1 Y76A^ under all of the conditions tested, confirming that the mutant protein was still able to repress the island but was insensitive to the Sri inducer (Figure 7C). Finally, we tested the impact of the Stl^SaPI1 Y76A^ mutation *in vivo*. We generated a SaPI1 *tst*::*tet*M derivative island expressing Stl^SaPI1 Y76A^. Note that this island carries an antibiotic resistance marker which facilitates transfer studies. The strain was then lysogenized with phage 80α, and the transfer of the island was analysed after induction. As a control, we included a strain lysogenic for 80α carrying the wt SaPI1 *tst*::*tet*M. Transfer of the SaPI1 mutant was significantly reduced compared to the wt SaPI1 (Figure 7D). Taken together, these results validate the Sri-Stl^SaPI1^ interactions revealed by the X-ray crystallographic data.

**Figure 8.**
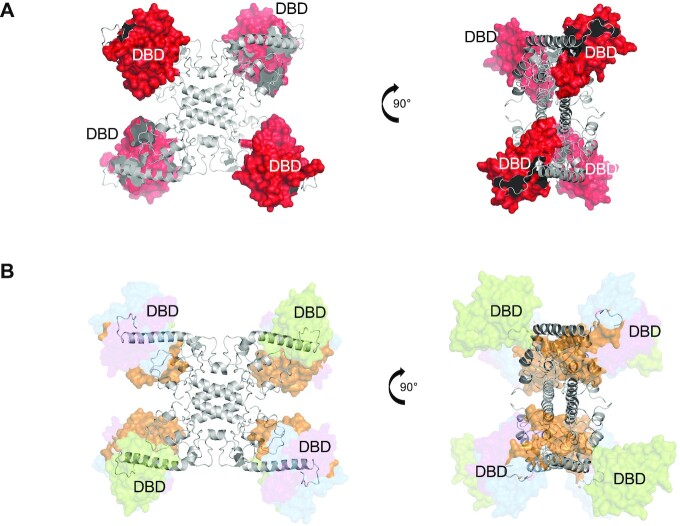
The Stl^SaPI1^ DBDs are flexible in solution. (**A**) Stl^SaPI1^ tetramer structure was generated by removing the Sri coordinates from the X-ray crystallography structure of the Stl^SaPI1^-Sri complex. Residues 90–244 are in grey and DBD surfaces are in red. (**B**) Model with the DBD conformers which best fit to the SAXS data via the ensemble optimization method for Stl^SaPI1^ in solution. Stl^SaPI1^ residues 90–244 are in grey and the DBD conformers are represented in surface with different transparencies proportional to the percentage of that model in the total ensemble. The fit was obtained with ∼ 50% of model 1 (DBDs in orange), 25% of model 2 (DBDs in green) and 13% of models 3 (DBDs in blue) and 4 (DBDs in purple).

Though we were unable to obtain the structure of Stl^SaPI1^ alone or complexed with its cognate DNA, we obtained low resolution structural information about this protein in solution using small-angle X-ray scattering (SAXS). First, we generated a SAXS data set for the Sri-Stl^SaPI1^ complex and for Stl^SaPI1^ alone merging 14 and 49 frames of SAXS data for which a constant *R*_g_ of 39.7 and 41.1 Å was estimated, respectively. The maximum particle dimension *D*_max_ was 127.3 and 139.1 Å for the Sri-Stl^SaPI1^ complex and for Stl^SaPI1^ alone, respectively (Supplementary Figure S8). The mass of a Sri-Stl^SaPI1^ monomer (including a His^6^ tag on Sri) is 38 997 Da, and the mass of Stl^SaPI1^ is 29 398 Da, which translates to masses of 155 988 Da for a tetramer of Sri-Stl^SaPI1^ or 117 592 Da for a tetramer of Stl^SaPI1^. Molecular weight analysis by Bayesian inference (22) in Primus estimated *M* = 124 450 Da (46.10% probability) with a credibility interval of [111 250, 134 300] (99.40% probability) for Stl^SaPI1^-Sri and *M* = 101 050 Da (32.44% probability) with a credibility interval of [92 650, 111 250] (95.59% probability) for Stl^SaPI1^ alone. The SAXS analysis was therefore strongly suggestive of a tetramer for both Sri-Stl^SaPI1^ and Stl^SaPI1^ alone. A model for a Stl^SaPI1^ tetramer was generated by removing the coordinates of Sri from the X-ray crystallography structure of the Stl^SaPI1^-Sri complex (Figure 8A). US-SOMO (35) was used to compute *R*_g_ = 36.8 Å for this model, indicating that the model extracted from the crystal structure is more compact than the Stl^SaPI1^ tetramer in solution. Thus, the mobility of the Stl^SaPI1^ DNA-binding domains (DBDs) was modelled using EOM (23) (Figure 8B, Supplementary Figure S8). 10 000 models were generated in which the C-terminal domains (CTD, residues 101–247) were kept in the conformation observed by X-ray crystallography but the DBDs (residues 1–89) were allowed to adopt positions consistent with their connection to the CTD via a native-like flexible linker (residues 90–100). In support of this, the crystallographic structure of Sri-Stl^SaPI1^ showed high mobility in the Stl^SaPI1^ DBDs when Sri was weakly bound (subunits B and B*, Figure 1B), as well as differences between subunits in the dimer (Figure 1B), supporting the notion that the structure of these DBDs is highly plastic in the absence of Sri and is compatible with binding to operators. The disposition of the Stl^SaPI1^ DBD was more extended compared with the DBD of the complex Sri-Stl^SaPI1^, which was also shown in our SEC-MALS results where a higher hydrodynamic volume was observed for Stl^SaPI1^ than for the Sri-Stl^SaPI1^ complex (Supplementary Figure S1).

These results indicate that SaPI1 de-repression involves a mechanism different from that used by SaPIbov1. While the latter involves separation of the Stl^SaPIbov1^ dimer by the inducing dUTPases (9), our data show that Sri de-represses SaPI1 by inducing a conformational change in the Stl^SaPI1^ DBDs, preventing the binding of these domains to their operators.

### Stl^SaPI1^ homologs are widespread in nature

**Figure 9.**
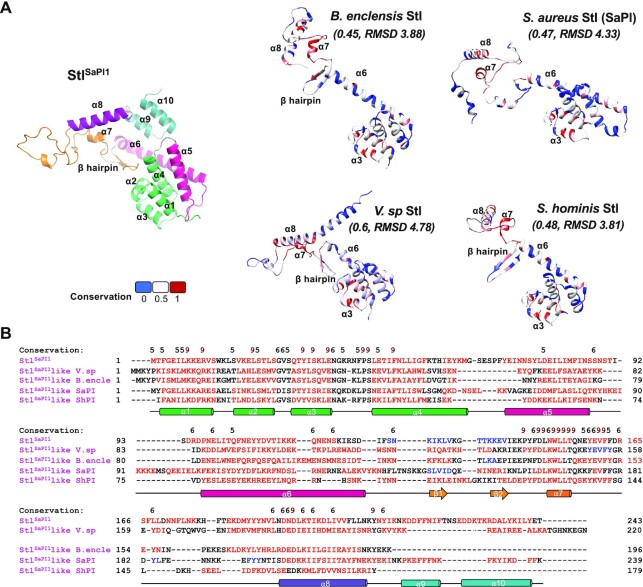
Stl^SaPI1^ repressor conservation in other species. (**A**) Stl^SaPI1^-like repressors present in other bacterial species were modelled using Alphafold server. The Stl^SaPI1^ monomer structure in the left part of panel A coloured as follows: the DBD is coloured in green, the helices ⍺5 and ⍺6 are in pink, the central part of the molecule (β hairpin, α7 and α7-α8 connection) is in orange. The helix α8 is in purple and the C-terminal part (helices α9 and α10) is in blue. The Stl^SaPI1^-like repressor models are represented and coloured based on their residue conservation: blue for non-conserved (0), red for conserved (1) and white for similar residues (0.5). The TM-scores and RMSD for the models compared with the Stl^SaPI1^ structure are indicated in italics in brackets. (**B**) Structural alignment of Stl^SaPI1^ and Stl^SaPI1^-like repressors. The residues forming the α helices or β strands are coloured in red and blue, respectively. The residue conservation amongst the different proteins is represented on the alignment (with 9 indicating conservation, and 5 similarity). The Stl^SaPI1^ secondary structure is represented below the alignment with colours defined in panel A.

Because of the unusual nature of the SaPI1 repression system, we wanted to know if it was exclusive to this island or more widespread in nature. In a search for Stl^SaPI1^-like homologs in the publicly accessible databases, different homologs were found in Staphylococci and different species of *Bacillus* and *Virgibacillus*, which have a sequence identity (compared to Stl^SaPI1^) that ranges from 26 to 35% (Supplementary Table S10). Importantly, while the Stl^SaPI1^-like homologs in *Staphylococcus* spp. were encoded by different members of the PICI family (Supplementary Table S10), suggesting a mechanism of induction in common with that reported here for SaPI1, the homologs present in the other genera were encoded by MGEs other than PICIs. Moreover, the 3D models obtained by the AlphaFold server for these proteins confirmed their structural homology with Stl^SaPI1^ (TM-scores 0.45–0.6; Figure 9A and Supplementary Figure S9), and the detailed analysis of the secondary structure of these homologs showed that the DBD (present in the first 3 α helices) and the dimerization (region between helices α7 and α8) or tetramerization (helix α8) key residues were extremely well conserved among these proteins (Figure 9B). These results confirm the discovery of a new family of repressors involved in gene transfer and bacterial evolution.

## DISCUSSION

Many MGEs, including prophages, PICIs or ICEs, control their life cycles by expressing master repressors that maintain these elements integrated into the bacterial chromosome. Importantly, the expression of these repressors must be precisely controlled since an excess of the repressor would impede the induction and transfer of the element, while a reduced expression would generate either the loss or the activation of the element under unfavourable conditions.

To ensure tightly-regulated control, SaPI1 has evolved a unique system involving two Stl^SaPI1^ tetramers and four operators. As previously mentioned, we proposed here two different models for SaPI1 repression (Figure 6). The best characterized repressor so far is CI from phage λ. CI represses both *cI* and *cro* expression by binding at operators *OL* and *OR*, each composed of three repressor binding sites named *OL1*, *OL2* and *OL3*, or *OR1*, *OR2* and *OR3*, respectively (36). Two CI dimers bind tightly and cooperatively to *OL1* and *OL2*, creating a tetramer that represses expression from the *pL* promoter (37). A similar tetramer bound structure is formed after the binding of two CI dimers to *OR1* and *OR2*, repressing in this case the expression from the *pR* promoter (37). To generate a more stable repression system, these two tetramers interact to form an octamer looping the DNA between the *OL* and *OR* operator regions (11,38). While Stl^SaPI1^ and CI repression involve the formation of tetramers, these two systems are completely different, both structurally and mechanistically, probably in response to the different ways that phages and SaPIs are induced. In λ, the two tetramers and the octamer appear only after the binding of the CI dimers to their cognate binding sites, while Stl^SaPI1^ is always a tetramer. Moreover, our results indicate that the binding of the two Stl^SaPI1^ tetramers to their cognate DNAs is not cooperative but sequential. Structurally, these two repressors are completely unrelated, except for their DBD regions. Functionally, they also work in completely different ways. Thus, after activation of the bacterial SOS response, the RecA* protein will promote the autocleavage of CI, disrupting dimer, tetramer and octamer formation, while in the case of SaPI1, the Sri protein does not affect tetramerization of Stl^SaPI1^ but will force the Stl^SaPI1^ DBDs to adopt a conformation that prevents their interaction with their cognate DNA boxes.

Two other systems involving tetramer formation have been described in the control of the transfer of different MGEs. The repressor (Rep) of temperate *Salmonella* phage SPC32H can reversibly assemble into two oligomeric states (dimer and tetramer) in a concentration dependent manner (39). As with Stl^SaPI1^, Rep binds to DNA as a tetramer, though these tetramers are structurally different. Contrary to what we observed with the Stl^SaPI1 L201E^ dimer, the dimeric Rep protein binds DNA weakly even at high concentrations. This difference can be easily explained since it has been proposed that the dimer pairs required for binding to the palindromic DNA sites originate from different dimers in the tetrameric Rep (39), while in the Stl^SaPI1^ they come from the same dimer. Another difference between these systems relates to how they are de-repressed. While the SaPI1 inducer Sri is a monomer that forces a conformational change in the Stl^SaPI1^ DBDs, the SPC32H anti-repressor Ant is a tetramer that binds to two dimeric Reps, breaking tetramer formation. Another important difference between the two systems is that only one tetramer is required for phage SPC32H repression (39), but two are required for SaPI1 repression.

The second system is from the *Enterococcus faecalis* conjugative plasmid pCF10. This plasmid encodes PrgX, which is a repressor that blocks the expression of genes involved in the conjugative transfer of this plasmid. As with Stl^SaPI1^, PrgX is a tetramer that binds to two different operator regions and forces a looping of the DNA (40). However, Rep, PrgX and Stl^SaPI1^ are unrelated in structure. Moreover, the mechanism involving pCF10 transfer is also different from that observed for SaPI1. The transfer of the pCF10 plasmid occurs in response to an intracellular pheromone signal, a peptide called cCF10 with the sequence LVTLVFV. As with Rep, binding of the cCF10 inducer to PrgX destabilizes the PgrX tetramer and promotes conjugation (40).

The new repression mechanism described here for SaPI1 is possible due to the localization of the Stl^SaPI1^ DBDs in the tetramer. Canonical members of the HTH-XRE family of repressors dimerize through their α5 helix. In Stl^SaPI1^, the α5 helix connects the DBDs with the rest of the protein by a long loop which confers high mobility for DNA recognition and binding (Figure 1C). The flexibility of the Stl^SaPI1^ DBDs was obvious when we compared the four DBD domains in the Sri-Stl^SaPI1^ X-ray crystallographic structure versus the SAXS data for Stl^SaPI1^. By interacting with Stl^SaPI1^ DBD helices α1, α4 and α5 and two other subunits in the tetramer, Sri maintains the Stl^SaPI1^ DBDs fixed in a conformation that prevents their binding to operators. However, in the absence of Sri, the DBDs showed a more extended localization that allow them to interact with their cognate DNA boxes (Supplementary Figure S7). This plasticity in Stl^SaPI1^ provides conformational freedom to the DBDs and is likely to be the origin of our inability to obtain X-ray structure crystallographic structures for Stl^SaPI1^ and Stl^SaPI1 L201E^.

Another interesting feature of the regulatory system is that the primary role of the phage-encoded SaPI1 inducer is not to induce the island but to interact with the cellular DnaI protein (34), slowing down bacterial replication and facilitating phage reproduction. Thus, SaPI1 has evolved a repressor that uses a conserved phage protein as its inducer. Since DnaI and Stl^SaPI1^ are completely unrelated in sequence and structure, how this small protein interacts with two unrelated proteins to perform two different functions increases the interest of this system and is currently under study. Other interesting unsolved questions include what the origin of this repressor is, and how SaPI1 co-opted and evolved it to adapt to its life cycle requirement. While we cannot answer these questions yet, it is clear that this repressor is not unique to SaPI1 but is present in other MGEs from different species. It is also clear the SaPIs have evolved an impressive arsenal of strategies to hijack the life cycle of their helper phages, making these elements one of the most sophisticated subcellular parasites in nature.

## DATA AVAILABILITY

Atomic coordinates and structure factors have been deposited at the RCSB Protein Data Bank (PDB) (PDB code 7P4A for Sri-Stl^SaPI1^ and 1ZVI for Sri-Stl^SaPI1 L201E^).

## Supplementary Material

gkac855_Supplemental_Files

## Notes

Present address: Jason A. Tan. Department of Biology, Augsburg University, Minneapolis, MN 55454, USA.
