# Supplementary material for: Non-canonical Staphylococcus aureus pathogenicity island repression
Source: Nucleic Acids Res. 2022 Oct 6;50(19):11109–27. doi: 10.1093/nar/gkac855 (PMC9638917; doi:10.1093/nar/gkac855)
Supplement: gkac855_Supplemental_Files [file gkac855_supplemental_files.zip › Supplementary Figures.pdf]

A

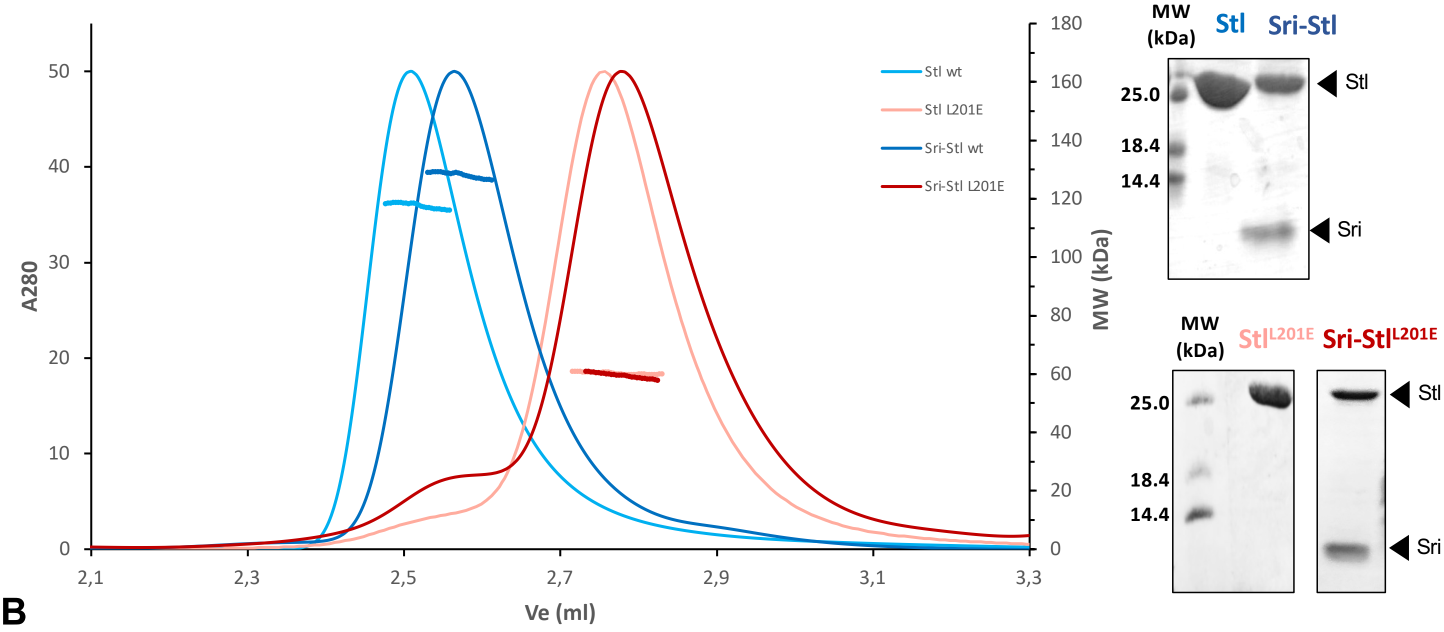

B

Protein	Theoretical Complex		Observed complex SEC-MALS	
	MW (kDa)	Complex	MW (kDa)	Complex
**Stl^{SaPI1} wt**	119.812	4 Stl	117.410 ± 0.197%	4 Stl
**Sri-Stl^{SaPI1} wt**	144.516	4 Sri : 4 Stl	127.589 ± 0.236%	1-2 Sri : 4 St
**Stl$^{SaPI1\ L201E}$**	59.938	2 Stl	59.455 ± 0.751%	2 Stl
**Sri-Stl$^{SaPI1\ L201E}$**	72.290	2 Sri : 2 Stl	60.410 ± 0.257%	0-1 Sri : 2 Stl

Figure S1. Stl^{SaPI1} oligomeric state.

(A) The UV and MW data obtained in SEC-MALS are represented. Wild type Stl^{SaPI1} is colored in blue, while Stl$^{SaPI1\ L201E}$ is in red, both alone (light colors) or in complex with Sri (dark colors). Samples obtained from the elution peaks of each sample were run in a 17% SDS-PAGE (right).

(B) Table with the SEC-MALS results for the wt Stl^{SaPI1} and the Stl$^{SaPI1\ L201E}$ proteins, both alone or in complex with Sri. Theoretical and observed MW are represented.

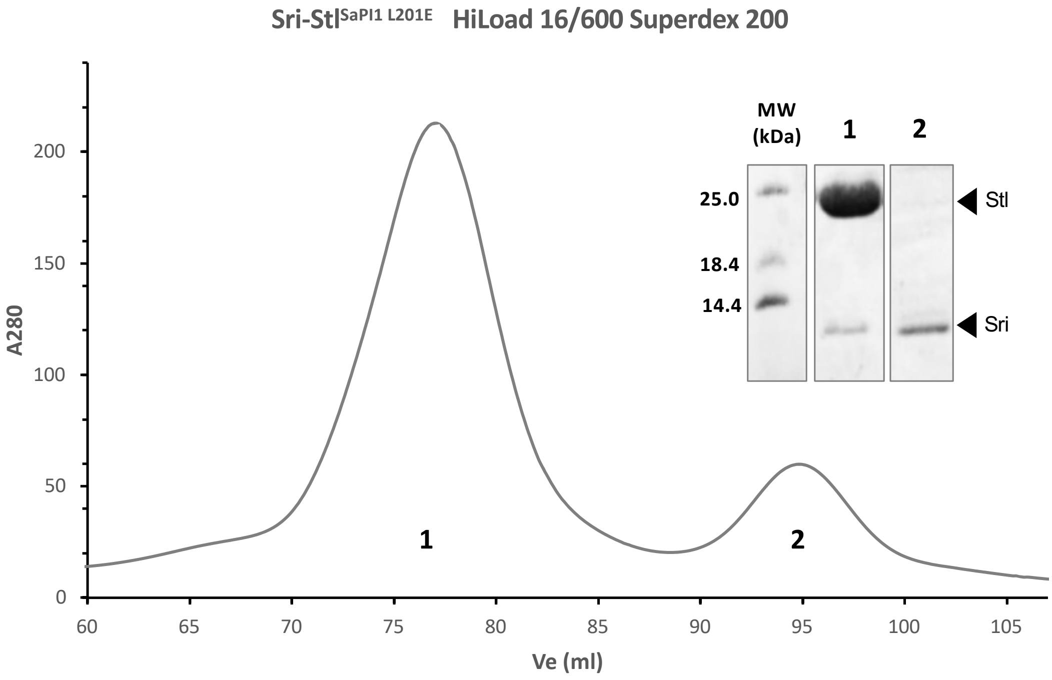

Figure S2. Size exclusion chromatography of the Sri-Stl^{SaPI1} complex.

Size exclusion chromatography in HiLoad 16/600 Superdex 200 column with the Sri-Stl^{SaPI1} complex. Samples collected from peak 1 and 2 were analyzed in 17% SDS PAGE.

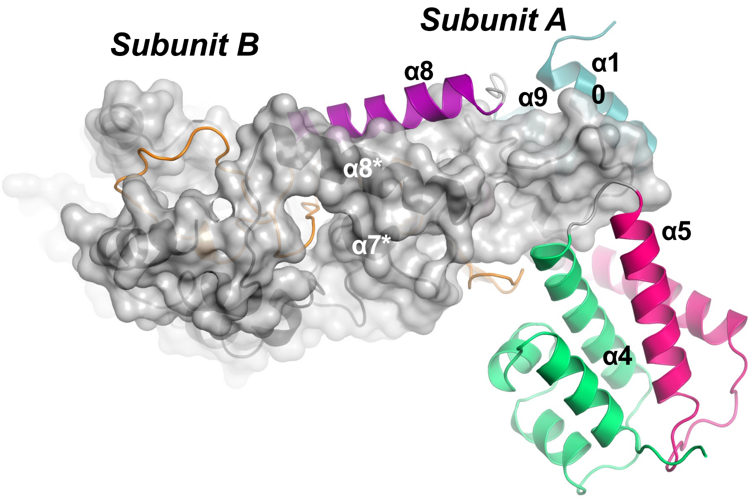

Figure S3. Structures of the Stl^{SaPI1} monomer and Stl^{SaPI1} dimer.

Details of the Stl^{SaPI1} dimerization in the asymmetric unit of the crystal. One Stl^{SaPI1} molecule (subunit A) is colored as follow: the DBD is colored in green, the helices α5 and α6 are in pink, the central part of the molecule (β hairpin, α7 and α7- α8 connection) is in orange, the helix α8 is in purple and the C-terminal part (helices α9 and α10) is in blue. The second molecule of the dimer (subunit B) is represented in grey and surface. Helices α4, α5, α9 and α10, plus the β harpin of subunit A create a cavity where the connector helices α7* and α8* from subunit B are located.

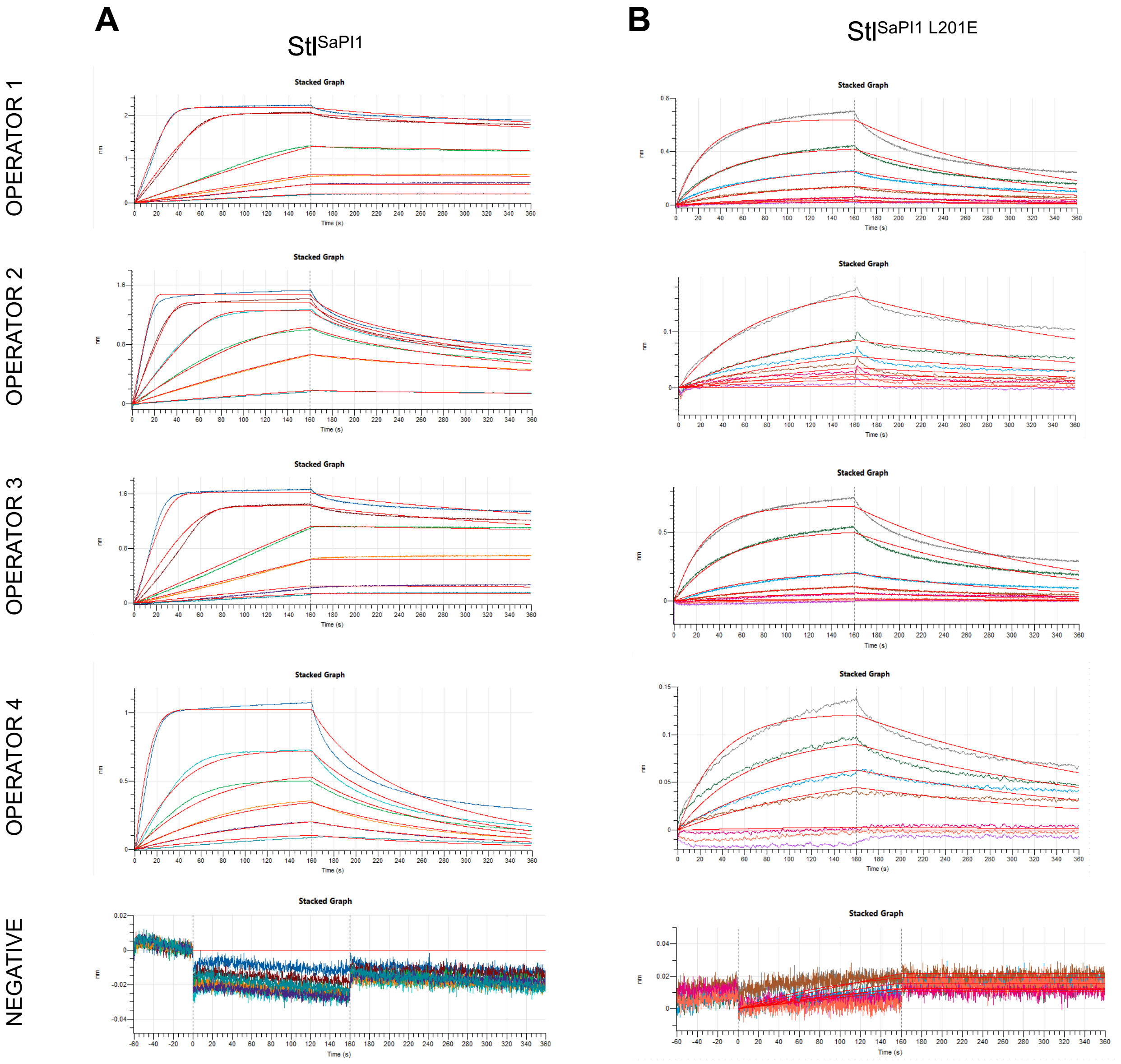

Figure S4. Bilayer interferometry kinetic curves of Stl^{SaPI1} with their cognate operator sites

(A) Stl^{SaPI1} binding curves for the individual four operators and the negative DNA probe. The 100 mM protein concentration curve is colored in dark blue, the 50 nM in brown, 25 nM in cyan, 12.5 nM in green, 6.25 nM in orange, 3.125 nM in purple and the 1.562 nM in dark cyan. The fitting was adjusted to a mass transport model and the fitting curves are represented in red.

(B) as in (A) for the Stl$^{SaPI1\ L201E}$ protein. The fitting was adjusted to a 1:1 binding model.

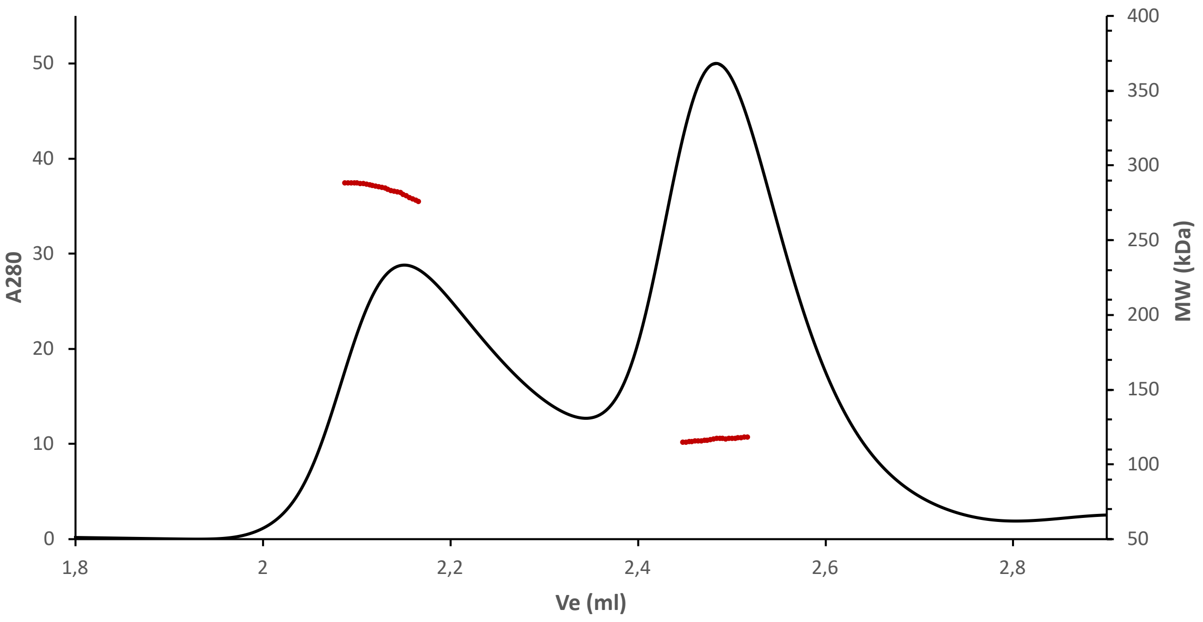

		Operator		Loaded ratio (protein-DNA)	Teorical Complex		Observed complex MALS		
	MW (kDa)		MW (kDa)		MW (kDa)	Complex	Peak	MW (kDa)	Complex
**Stl^{SaPI1} wt tetramer**	119.812	**-**	-	-	119.812	4 Stl		117.410	4 Stl
		**1-4**	70.11	4 tetramers: 1 DNA	549.358	8 Stl : 1 DNA	1	283.482	8 Stl : 1 DNA
							2	116.785	4 Stl

Figure S5. Stl^{SaPI1} DNA binding characterization by SEC-MALS

The UV (black line) and MW (red dots) data obtained in SEC-MALS for a sample containing the DNA probe with the four operators and an excess of 4-fold molar ratio of Stl^{SaPI1}. The first peak corresponds to the Stl^{SaPI1} bound to the DNA and the second with the excess of Stl^{SaPI1}. The calculated MW is collected in the table. The MW of Stl^{SaPI1} obtained by SEC-MALS was also added to the table.

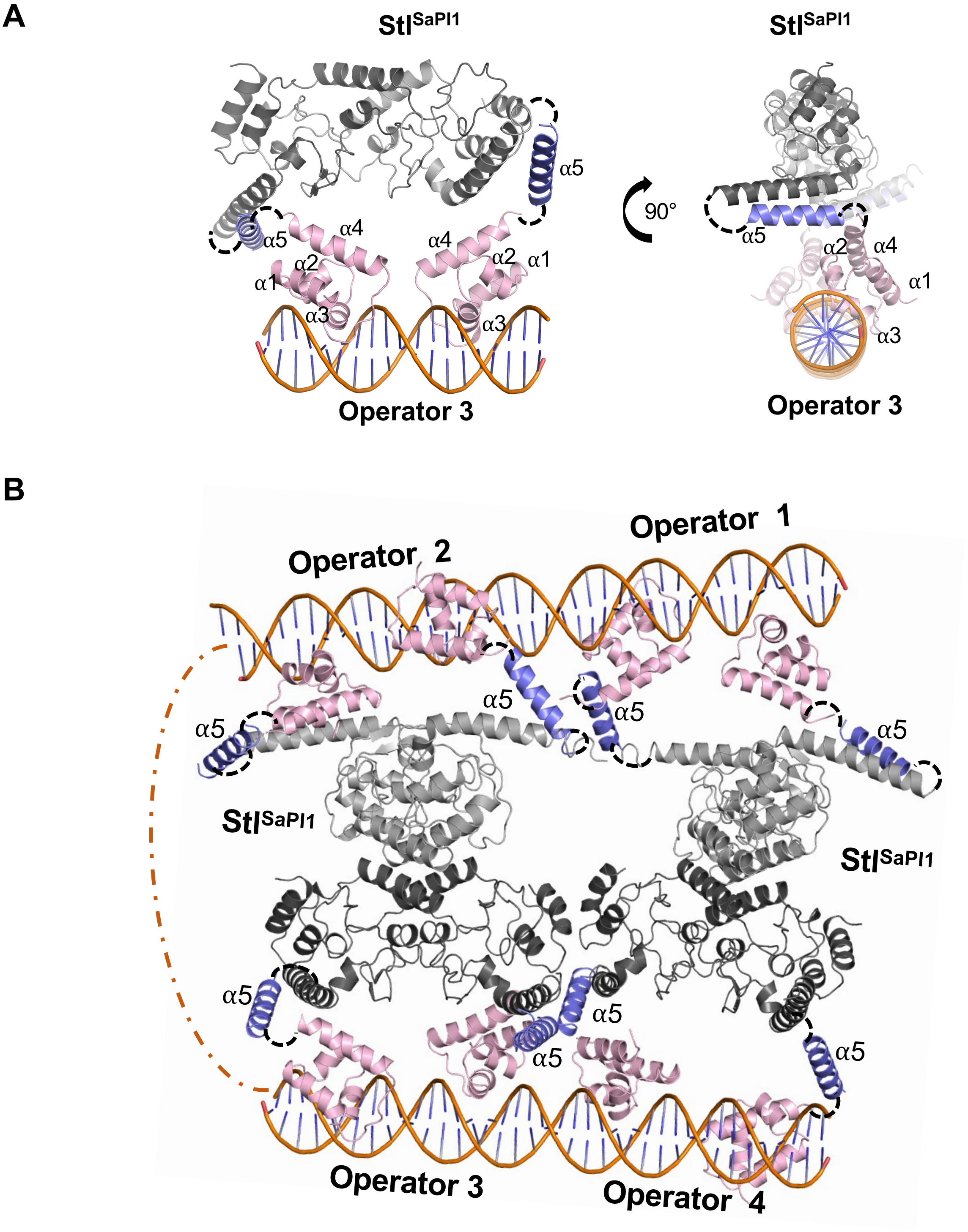

Figure S6. Stl^{SaPI1} repression models.

(A) Model of Stl^{SaPI1} dimer bound to the operator 3. The central part of the protein dimer is in grey and the DBDs in pink. The helix α5 is colored in purple and the flexible loops, which connect it to helixes α4 and 6, are represented in dashed lines showing its role as a connector structural element.

(B) Model of the two Stl^{SaPI1} tetramers bound to the *stl-str* intergenic region. Stl^{SaPI1} is colored as in (A). The operators are labelled and the torsion between the operators 2 and 3 is represented as orange dashed lines.

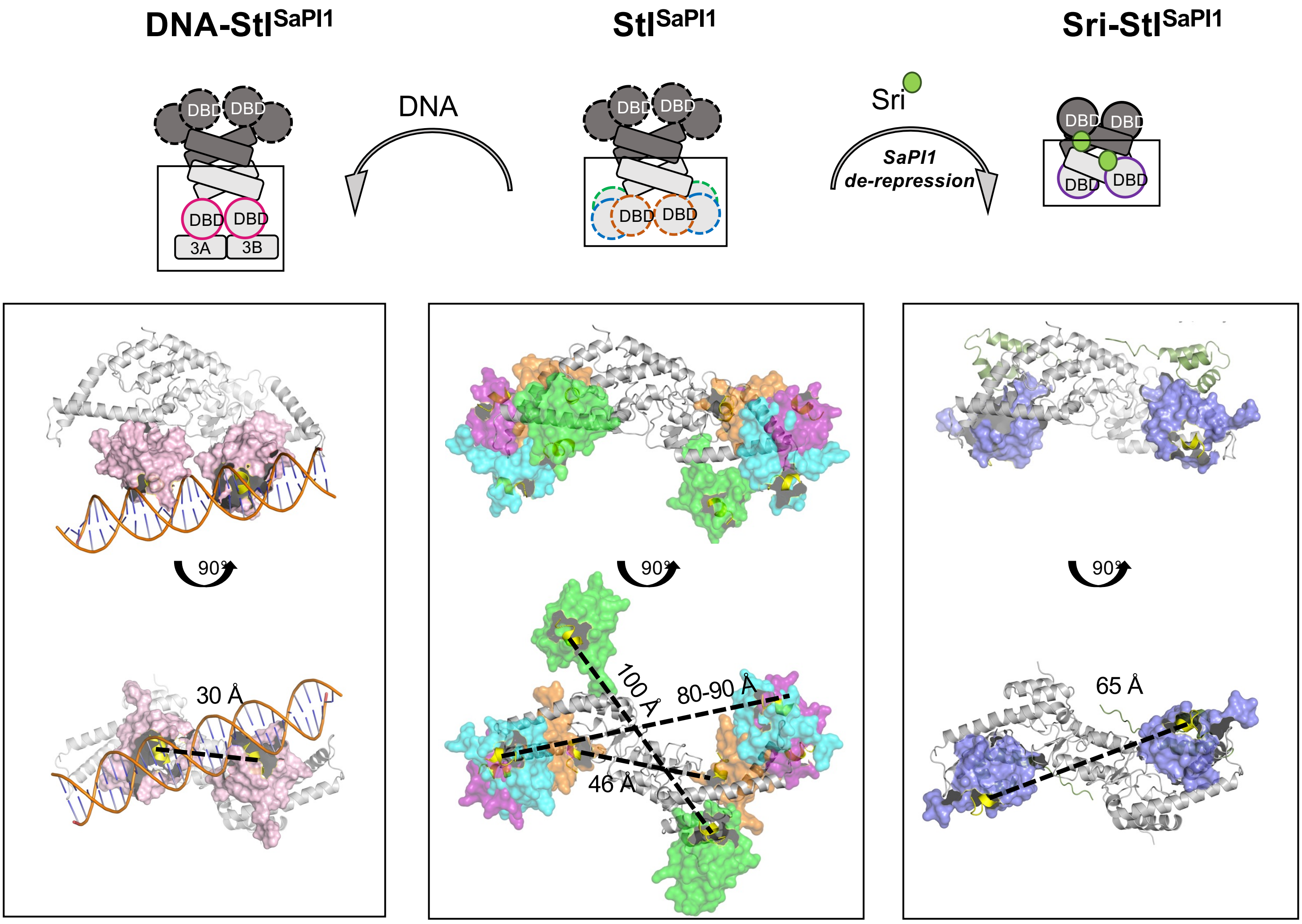

Figure S7. Stl^{SaPI1} DBD flexibility.

Model of the Stl^{SaPI1} DBDs location when the repressor is bound to its operator regions repressing SaPI1 (left). Model obtained by SAXS of Stl^{SaPI1} DBDs location in solution (middle). X-ray structure of Sri bound to Stl^{SaPI1} derepressing SaPI1 (right). The DBDs are represented as surface in pink for Stl^{SaPI1}-DNA; orange, cyan, green and purple when Stl^{SaPI1} is free and in blue for Stl^{SaPI1}-Sri complex. Sri is represented in green. The DBD α3 helix responsible for the interaction with the DNA is represented in cartoon in yellow. The distance between α3 helices in the Stl^{SaPI1} dimer is indicated in the figure.

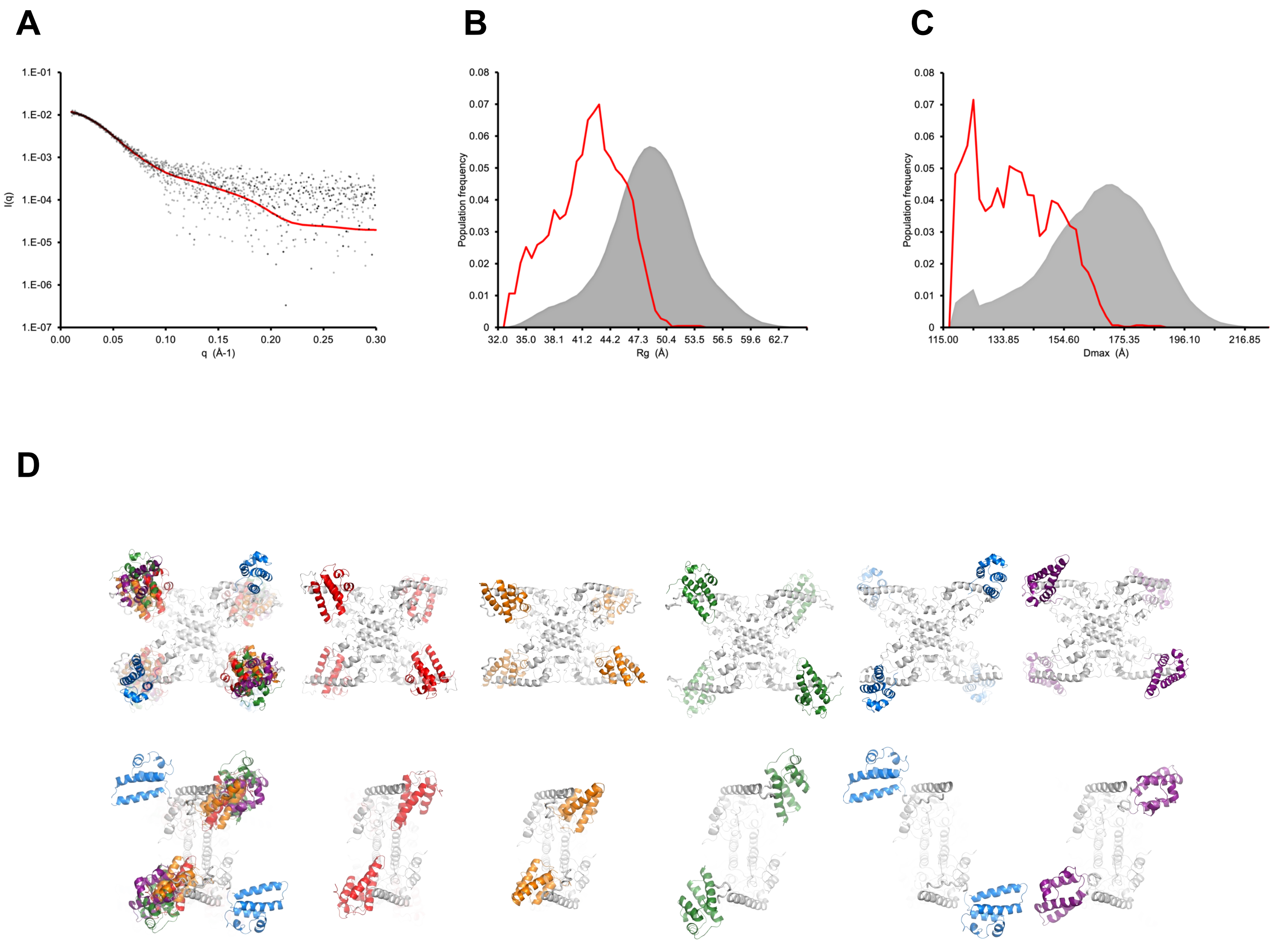

Figure S8. Analysis of Stl^{SaPI1} tetramer conformational heterogeneity and solution ensembles.

(A) Overlay of experimental SAXS data for Stl^{SaPI1} (dots) and those computed for the best-fitting ensemble selected by EOM with χ^2 = 1.31 (red line).

(B) R_g and D_{max} (C) computed for a pool of 10000 solution conformers (grey) compared with that computed for the best-fitting ensemble generated by EOM (red) for Stl^{SaPI1} tetramer in which the C-terminal domains were kept in the conformation observed by X-ray crystallography but the N-terminal DNA-binding domains were allowed to adopt positions consistent with their connection to the CTD via a native-like flexible linker.

(D) Orthogonal views of the superimposition upon a model for the Stl^{SaPI1} tetramer of 4 Stl^{SaPI1} conformers that combined provide the best fit to the SAXS data via the EOM. The fit was obtained with ~ 50% of model 1 (DBDs in orange), 25% of model 2 (DBDs in green), and 13% of models 3 (DBDs in blue) and 4 (DBDs in purple). DBDs of the Stl^{SaPI1} tetramer model are in red.

S. aureus Stl (SaPI)

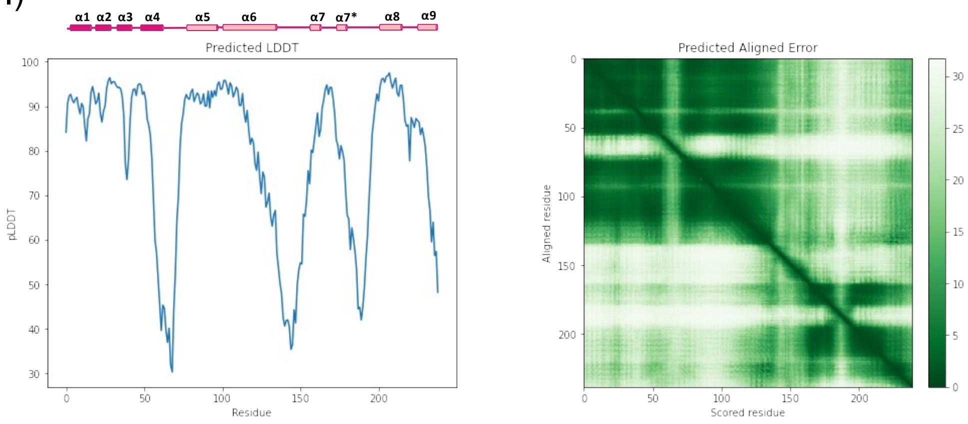

S. hominis Stl

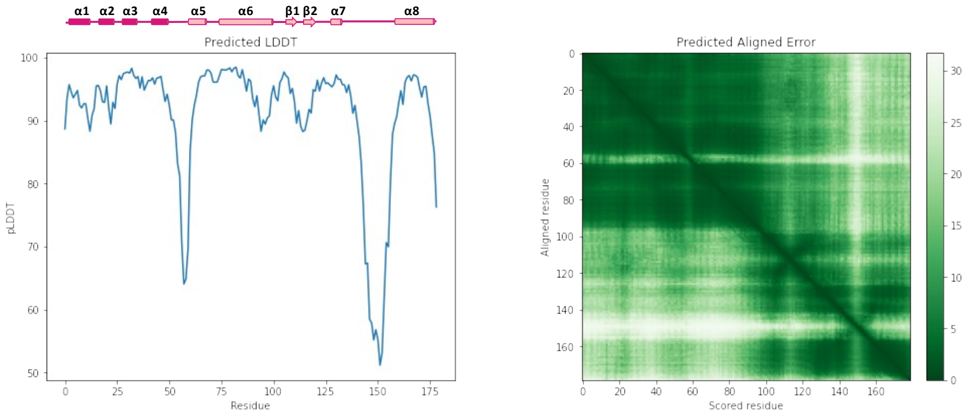

V. sp Stl

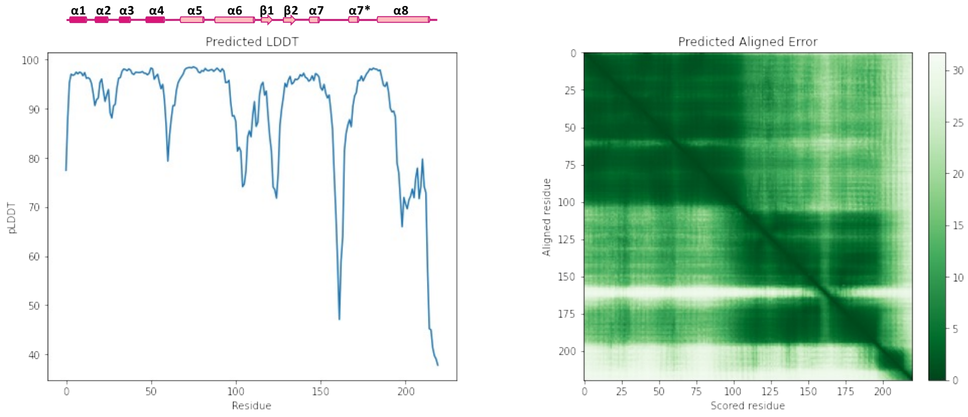

B.encensis Stl

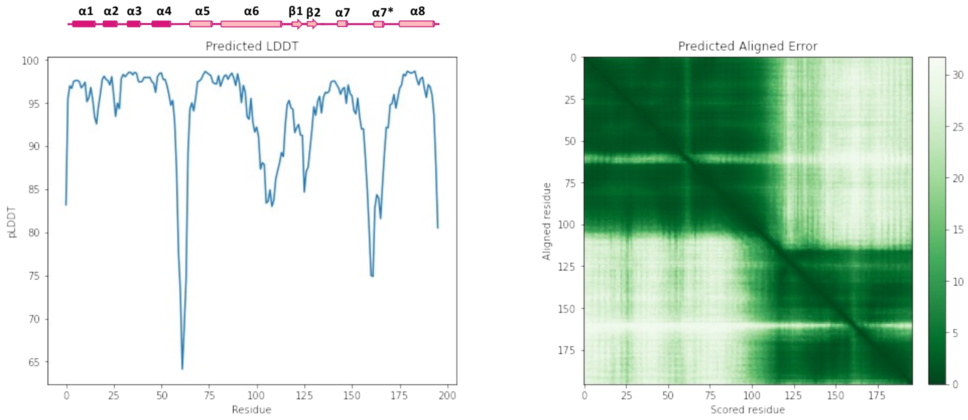

Figure S9. Quality of the Stl like models obtained with Alphafold server.

pLDDT residues representation and Predicted Alingment Error plot for the *S. aureus, S. hominis, Virgibacillus* spp. and *B. enclensis* Stl like protein models obtained with Alphafold server. The secondary structure of the models are represented in pink on the top of the pLDDT graph.
