# Supplementary material for: Non-canonical Staphylococcus aureus pathogenicity island repression
Source: Nucleic Acids Res. 2022 Oct 6;50(19):11109–27. doi: 10.1093/nar/gkac855 (PMC9638917; doi:10.1093/nar/gkac855)
Supplement: gkac855_Supplemental_Files [file gkac855_supplemental_files.zip › Tables_Supple.docx]

**Table S1. Bacterial strains used in this work.**

| **Stock** | **Description** | | **Reference** |
| --- | --- | --- | --- |
| DH5α | *E. coli* host for DNA cloning | | Invitrogen |
| RN4220 | *S. aureus* restriction-defective derivate of RN450 | | ^3^ |
| BL21 (DE3) | *E. coli* expression strain | | Stratagene |
| JP19607 | *E. coli* DH5α pJP2371 | | This work |
| JP22989 | *E. coli* DH5α pJP2700 | | This work |
| JP709 | *E. coli* DH5α pJP2344 | | This work |
| JP17789 | *E. coli* DH5α pJP2366 | | This work |
| JP17792 | *E. coli* DH5α pJP2369 | | This work |
| JP17813 | *E. coli* DH5α pJP2387 | | This work |
| JP17816 | *E. coli* DH5α pJP2390 | | This work |
| JP2799 | RN10359 lysogenic for wt 80α | | This work |
| JP15519 | JP2799 SaPI1 | | This work |
| JP17848 | RN4220 Y76A Stl SaPI1 | | This work |
| JP19902 | RN10359 lysogenic for wt 80α + Y76A Stl SaPI1 | | This work |
| JP17863 | RN4220 pJP2387 | | This work |
| JP17866 | RN4220 pJP2390 | | This work |
| JP17891 | JP2799 pJP2387 | | This work |
| JP17894 | JP2799 pJP2390 | | This work |
| JP15526 | BL21 (DE3) pJP2344 | | This work |
| JP17839 | BL21 (DE3) pJP2366 | | This work |
| JP17842 | BL21 (DE3) pJP2369 | | This work |
| JP23638 | BL21 (DE3) pJP2371 | | This work |
| JP17873 | RN4220 pCN41 | | This work |
| JP17901 | JP2799 pCN41 | | This work |
| JP22095 | *E. coli* DH5α pJP2699 | | This work |
| JP22101 | JP2799 pJP2699 | | This work |
| JP23508 | *E. coli* DH5α pJP2701 | | This work |
| JP23523 | RN4220 pJP2701 | | This work |
| JP23531 | JP15519 pJP2701 | | This work |
| JP23534 | *E. coli* DH5α pJP2702 | | This work |
| JP23536 | RN4220 pJP2702 | | This work |
| JP23640 | JP15519 pJP2702 | | This work |
|  | |  |  |
|  |  | |  |

**Table S2. Plasmids used in this work.**

| **Plasmid** | **Description** | **Reference** | | |
| --- | --- | --- | --- | --- |
| pPROEX-Hta | Expression vector | | Invitrogen |  |
| pCN41 | Expression vector | | ^5^ |  |
| pJP2700 | pPROEX Hta His-Stl SaPI1 | | This work |  |
| pJP2344 | pPROEX Hta His- Sri 80α + Stl SaPI1 | | This work |  |
| pJP2366 | pPROEX Hta His- Sri 80α + Y76A Stl SaPI1 | | This work |  |
| pJP2369 | pPROEX Hta His- Sri 80α + L201E Stl SaPI1 | | This work |  |
| pJP2371 | pPROEX Hta His- L201E Stl SaPI1 | | This work |  |
| pJP2384 | pCN41- Stl-Str region of SaPI1 | |  |  |
| pJP2387 | pCN41- (Y76A Stl)-Str region of SaPI1 | | This work |  |
| pJP2390 | pCN41- (L201E Stl)-Str region of SaPI1 | |  |  |
| pJP2699 | pCN41- Stl-Str region of SaPI1 with P*str* -35 mutant (TTGACA to TTAACA) | | This work |  |
| pJP2806 | pCN41- stl promotor of SaPI1 | | This work |  |
| pJP2807 | pCN41- stl promotor of SaPI1 with P*stl* -35 mutant (AGTACA to AGTAAA) | | This work |  |

**Table S3. Primers used in this work.**

| **Clone** | **Oligonucleotide** | **Sequence (5'-3')** | |
| --- | --- | --- | --- |
| pJP2344 | orf22-phi80a-6mB | CGCGGATCCGATGGTAACCAAAGAATTTTTG | |
|  | ORF22-80a-12cE | CCGGAATTCCTTTCGGCATTTTTAACACTCCTTAATATTCGACGATAGCGGGGC | |
|  | E11-STL1-35mE | CCGGAATTCTTCACACAGGAAACAGACCATGATTTATATGACTTTTGGTGAAATATT | |
|  | SaPI1-49cS | ACGCGTCGACCATTAATCTGTTTCATATAAAATT | |
| pJP2366 | pPROEX-66m | GTTTGACAGCTTATCATCGAC | |
|  | pPROEX-69c | CTCTCATCCGCCAAAACAGC | |
|  | STL1-Y76A-32m | GAAATCAATAATAGTGCGTTAGATGAAATTCTC |  |
|  | STL1-Y76A-33c | GAGAATTTCATCTAACGCACTATTATTGATTTC | |
| pJP2369 | pPROEX-66m | GTTTGACAGCTTATCATCGAC | |
|  | pPROEX-69c | CTCTCATCCGCCAAAACAGC | |
|  | STL1-L201E-45m | TAAAGACAATTAAAGATGAAATCGTTGTATTTTTAC | |
|  | STL1-L201E-46c | GTAAAAATACAACGATTTCATCTTTAATTGTCTTTA | |
| pJP2371 | pPROEX-66m | GTTTGACAGCTTATCATCGAC | |
|  | pPROEX-69c | CTCTCATCCGCCAAAACAGC | |
|  | STL1-L201E-45m | TAAAGACAATTAAAGATGAAATCGTTGTATTTTTAC | |
|  | STL1-L201E-46c | GTAAAAATACAACGATTTCATCTTTAATTGTCTTTA | |
| pJP2384 | STL1-pCN41-55mE | CCGGAATTCACCGAACTGTATGAAATTC | |
|  | STL1-pCN41-56cB | CGCGGATCCGTATAAAAATTCCTGAGATTATC | |
| pJP2387 | STL1-pCN41-55mE | CCGGAATTCACCGAACTGTATGAAATTC | |
|  | STL1-pCN41-56cB | CGCGGATCCGTATAAAAATTCCTGAGATTATC | |
|  | STL1-Y76A-32m | GAAATCAATAATAGTGCGTTAGATGAAATTCTC | |
|  | STL1-Y76A-33c | GAGAATTTCATCTAACGCACTATTATTGATTTC | |
| pJP2390 | STL1-pCN41-55mE | CCGGAATTCACCGAACTGTATGAAATTC | |
|  | STL1-pCN41-56cB | CGCGGATCCGTATAAAAATTCCTGAGATTATC | |
|  | STL1-L201E-45m | TAAAGACAATTAAAGATGAAATCGTTGTATTTTTAC | |
|  | STL1-L201E-46c | GTAAAAATACAACGATTTCATCTTTAATTGTCTTTA | |
| pJP2699 | STL1-pCN41-55mE | CCGGAATTCACCGAACTGTATGAAATTC | |
|  | STL1-pCN41-56cB | CGCGGATCCGTATAAAAATTCCTGAGATTATC | |
|  | Str-35-32m | CAATATTTTTTGTGTTAACAATACAAAAAGAAG | |
|  | Str-35-33c | CTTCTTTTTGTATTGTTAACACAAAAAATATTG | |
| pJP2702 | STL1-pCN41-3m | CCGCCTTTGAGTGAGCT | |
|  | STL1-pCN41-2c | CATGCACTTAAAACTAAAGC | |
|  | Stl-35-28m | GTTGTACTTTGAGTAAAATATTTTTTGTGTTGAC | |
|  | Stl-35-35c | ATCCAAAAAATATTTTACTCAAAGTACAA | |

**Table S4. Sri-Stl^SaPI1^ anomalous data peak intensities.**

| **RESIDUE (CHAIN)** | **COORDINATES** | | | **PEAK HEIGHT (SIGMA)** |
| --- | --- | --- | --- | --- |
|  | **X** | **Z** | **Y** |  |
| MSE 184 (B) | 67.886 | 71.254 | 24.531 | 18.75 |
| MSE 63 (A) | 60.881 | 84.687 | 40.434 | 15.08 |
| MSE 1 (A) | 72.251 | 80.950 | 57.337 | 13.31 |
| MSE 83 (A) | 74.137 | 83.107 | 49.731 | 8.68 |
| MSE 184 (A) | 21.573 | 50.625 | 39.410 | 7.69 |
| MSE 16 (E) | 71.698 | 86.983 | 47.942 | 5.89 |
| MSE 63 (B) | 21.905 | 50.223 | 17.585 | 5.34 |
| MSE -2 (A) | 78.472 | 90.219 | 61.811 | 5.02 |
| MSE 83 (B) | 16.152 | 29.944 | 20.042 | 4.15 |

**Table S5. Primers used for *str* and *stl* promotors characterization**

| **Promotor** | **Oligonucleotide** | **Sequence (5'-3')** |
| --- | --- | --- |
| *str* | SP1_5RACE-stl_Fw | CTTTAACCAATTTGATTTTATTACTAAATATATC |
|  | SP2_5RACE-stl_Fw | GTAATAAGTTCATTGGGGTCTCTATC |
|  | SP3_5RACE-stl_Fw | GTTTATAAACATTATGAGAATTTCATCTAAG |
| *stl* | SP1_5RACE-str_Fw | GTATGAAATTCCAAAAATACCAGC |
|  | SP2_5RACE-str_Fw | CATTAGGTTTCGCAAACAAC |
|  | SP3_5RACE-str_Fw | GTTCTGTAACTGTATTTTCTTTTAATGG |

**Table S6. Primers used for the EMSA and the bilayer Interferometry assays.**

| **Clone** | **Oligonucleotide** | **Sequence (5'-3')** |
| --- | --- | --- |
| Operator 1 | operator1_Fw | GGAGTTGTACTTTGAGTACAATACC |
|  | operator1_Rw | GGTATTGTACTCAAAGTACAACTCC |
| Operator 2 | Operator2_Fw | GGTTTTTGTGTTGACAATACAAACC |
|  | Operator2_Rw | GGTTTGTATTGTCAACACAAAAACC |
| Operator 3 | Operator3_Fw | GGTTTTGTACTCAATGTACAAAACC |
|  | Operator3_Rw | GGTTTTGTACATTGAGTACAAAACC |
| Operator 4 | Operator4_Fw | GGAAAAGTACTTGTAGTACTAAACC |
|  | Operator4_Rw | GGTTTAGTACTACAAGTACTTTTCC |
| Operator 1-4 | Operator1-4_Fw | GGATAAGTTGTACTTTGAGTACAATATTTTAAAAAATGAAAAAAAAAAAAGAAGTGTGTAATATTTGTTTAAAAAACAAAAAAAAAAAAAGTACTTGTAGTACTAAAAGAAC |
|  | Operator1-4_Rw | GGTTCTTTTAGTACTACAAGTACTTTTTTTTTTTTTGTTTTTTAAACAAATATTACACACTTCTTTTTTTTTTTTCATTTTTTAAAATATTGTACTCAAAGTACAACTTATCC |
| NEGATIVE | Operator3_NEG_Fw | GGTTTAAAAAAAAAAAAAAAAAACC |
|  | Operator3_NEG_Rw | GGTTTTTTTTTTTTTTTTTTAAACC |

**Table S7. Stl^SaPI1^ dimer contacts.**

| **SUBUNIT A** | | | **SUBUNIT B** | | | **Dist (Å)** |
| --- | --- | --- | --- | --- | --- | --- |
| **⍺4** | E60 | CD | **⍺7-⍺8 connec.** | T180 | CG2 | 3.6 |
| **⍺4-⍺5 connec.** | S65 | OG |  | D183 | OD2 | 3.5 |
|  | E66 | CA |  | K176 | CG | 3.8 |
| **⍺5** | Y70 | CD2 |  | H178 | CD2 | 3.6 |
| **⍺6** | Q116 | O |  | Y185 | OH | 3.9 |
|  | N119 | CB |  | Y185 | CE2 | 3.5 |
| **⍺6-β1 connec.** | F127 | CE1 |  | R165 | CG | 3.4 |
|  | N129 | CG |  | F162 | CB | 3.9 |
| **β1** | I131 | CD1 |  | F162 | CZ | 3.5 |
|  |  |  |  | Y186 | CE1 | 3.3 |
|  | K132 | CG |  | Y159 | CE1 | 3.2 |
|  | L133 | CD2 |  | E160 | CG | 3.6 |
|  |  |  |  | Y186 | CD1 | 3.9 |
| **β1-β2 connec.** | K135 | CD |  | N157 | C | 3.9 |
|  |  | NZ |  |  | OD1 | 2.8 |
|  | G136 | N |  | E160 | OE2 | 2.9 |
|  | T138 | CB |  | E181 | CD | 3.8 |
|  |  | OG1 |  |  | OE1 | 3.3 |
|  | F148 | CZ | **β2-⍺7 connec.** | Y147 | CE1 | 3.6 |
|  |  | CD1 | **⍺7** | L153 | CD1 | 3.7 |
|  |  | CZ | **⍺7-⍺8 connec.** | Y159 | CD2 | 3.6 |
|  | D149 | CG |  | F163 | C | 3.9 |
|  |  | OD2 |  |  | O | 3.2 |
| **⍺7** | L150 | CD1 | **⍺7** | L150 | CD1 | 3.3 |
|  |  | CA |  | L153 | CD2 | 3.8 |
|  |  | CD2 |  | L154 | CD2 | 3.9 |
|  |  | CB | **⍺7-⍺8 connec.** | V161 | CG2 | 3.9 |
|  |  | CG |  | F163 | CD1 | 3.8 |
|  |  | CD2 |  | L190 | CD1 | 3.8 |
|  |  | CD2 | **⍺8** | I198 | CD1 | 3.9 |
|  | N151 | CB | **⍺7-⍺8 connec.** | F163 | CE1 | 3.6 |
|  |  | CG |  | D164 | CG | 3.4 |
|  |  | OD1 |  |  | OD1 | 2.6 |
|  | L153 | CD1 | **⍺7** | F148 | CZ | 3.9 |
|  |  | CD2 |  | L150 | CA | 3.9 |
| **⍺7-⍺8 connec.** | Y159 | CZ | **β1** | K132 | CD | 3.6 |
|  |  | CE1 |  | V134 | CG2 | 3.7 |
|  |  | CD2 | **β2-⍺7 connec.** | F148 | CE2 | 3.3 |
|  | E160 | OE2 | **⍺4** | Y61 | OH | 3.7 |
|  |  | CG | **β1** | L133 | CD2 | 3.9 |
|  |  |  |  | V134 | C | 3.8 |
|  |  | CD |  | K135 | CA | 3.5 |
|  |  | OE1 |  | G136 | N | 3.7 |
|  | F162 | CD2 |  | N129 | CB | 3.9 |
|  |  | CZ |  | I131 | CG2 | 3.6 |
|  |  |  |  | L133 | CB | 3.6 |
|  |  | O | **⍺7** | D149 | OD1 | 2.8 |

| **SUBUNIT A** | | | **SUBUNIT B** | | | **Dist (Å)** |
| --- | --- | --- | --- | --- | --- | --- |
| **⍺7-⍺8 connec.** | F163 | CD1 | **⍺7** | L150 | CB | 3.9 |
|  |  | CE1 |  | N151 | CB | 3.4 |
|  |  |  | **⍺8** | I202 | CG2 | 3.7 |
|  |  | CZ |  | V203 | CG1 | 3.6 |
|  |  | CD2 |  | L206 | CD1 | 3.4 |
|  | D164 | CB | **⍺7** | N151 | CG | 3.7 |
|  |  | OD1 | **⍺9** | K216 | NZ | 2.9 |
|  |  | CG |  |  | CE | 3.7 |
|  | R165 | CZ |  | F220 | CE1 | 3.6 |
|  |  | NH2 | **⍺6-β1 connec.** | I126 | O | 2.8 |
|  |  | CD |  | F127 | CD1 | 3.4 |
|  |  | C | **⍺9** | K216 | CE | 3.4 |
|  | S166 | C | **⍺8 -⍺9 connec.** | Y212 | CD1 | 3.4 |
|  |  |  |  | I213 | CG1 | 3.6 |
|  | F167 | CE1 | **⍺8** | L206 | C | 3.8 |
|  |  |  |  | L207 | CD2 | 3.6 |
|  |  | CA | **⍺8 -⍺9 connec.** | Y212 | CD1 | 3.9 |
|  |  | C |  | I213 | CD1 | 3.6 |
|  | L168 | CB |  |  |  | 3.6 |
|  |  | CD2 | **⍺9** | F219 | CZ | 3.9 |
|  |  |  | **⍺10** | L240 | CD1 | 3.5 |
|  | L169 | CD1 | **⍺8 -⍺9 connec.** | Y210 | CD2 | 3.2 |
|  |  |  |  | N211 | CB | 3.7 |
|  |  | CD2 |  | I213 | CG2 | 3.7 |
|  |  |  | **⍺9** | F219 | CE2 | 3.8 |
|  |  | CD1 | **⍺10** | L240 | CD2 | 3.9 |
|  | D170 | CA | **⍺8 -⍺9 connec.** | Y210 | CE2 | 3.6 |
|  | N172 | CB |  |  | CD1 | 3.7 |
|  | F173 | CG | **⍺9** | F223 | CE1 | 3.7 |
|  |  | CE2 | **⍺10** | Y237 | CE1 | 3.6 |
|  |  |  |  | L240 | CD1 | 3.5 |
|  | L174 | O |  | Y237 | OH | 3.0 |
|  |  | CD1 |  | L240 | CD1 | 3.9 |
|  | F179 | O |  | D229 | OD2 | 3.6 |
|  |  | CD2 |  | K232 | CE | 3.9 |
|  |  | CE2 |  | R233 | CB | 3.7 |
|  |  | CZ |  | L236 | CD2 | 3.9 |
|  | K182 | CE | **β1** | T138 | CB | 3.8 |
|  | M184 | CB | **⍺9** | F223 | CG | 3.8 |
|  | Y185 | CD2 |  | T224 | CG2 | 3.5 |
|  | Y187 | CD2 |  | F223 | CD2 | 3.5 |
|  |  | OH |  | K216 | O | 3.9 |
|  |  | CE2 |  | F219 | CD2 | 3.7 |
|  |  |  | **⍺8** | I213 | CD1 | 3.7 |
|  | L190 | CD1 |  | L206 | CD2 | 3.9 |
| **⍺8** | D194 | OD1 |  | K209 | NZ | 2.7 |
|  |  | OD2 |  | Y210 | OH | 2.3 |
|  |  | CG |  |  | CZ | 3.9 |
|  | T197 | CG2 |  | F205 | CZ | 3.3 |

**Table S8. Stl^SaPI1^ tetramer contacts.**

|  | **Stl^SaPI1^ Subunit A** | **Stl^SaPI1^ Subunit A*** | **Stl^SaPI1^ Subunit B*** | **Dist. (Å)** |
| --- | --- | --- | --- | --- |
| **⍺8** | N193(OD1) |  | N208(OD1) | 3.6 |
|  |  |  | N208(ND2) | 2.8 |
|  | D194(OD1) |  | N208(OD1) | 3.6 |
|  | T197(CG2) |  | V204(CG1) | 3.8 |
|  |  |  | N208(CG) | 3.6 |
|  | T197(OG1) |  | N208 (ND2) | 2.9 |
|  |  |  | N208(OD1) | 2.5 |
|  | D200(CB) |  | V204(CG1) | 3.3 |
|  | L201(CD2) |  | L201(CD2) | 3.4 |
|  | L201(CG) |  | V204(CG1) | 3.8 |
|  | F205(CD1) | F205(CD1) |  | 3.9 |
|  | N208(CG) | F205(CZ) |  | 3.8 |
|  | K209(CG) | K209(CG) |  | 3.6 |

**Table S9. Sri-Stl^SaPI1^ contacts.**

| **Sri** | | **Stl^SaPI1^ Subunit A** | | **Stl^SaPI1^ Subunit A*** | | **Stl^SaPI1^ Subunit B** | | **Dist (Å)** |
| --- | --- | --- | --- | --- | --- | --- | --- | --- |
| α1-α2 connetion | E12(C) |  | MSE63(CE) |  |  |  |  | 3.7 |
|  | C13(CA) | α1 | W14(CE) |  |  |  |  | 3.8 |
| α2 | M16(CG) | α5 | Y76 (CE1) |  |  |  |  | 3.5 |
|  |  |  | I80(CD1) |  |  |  |  | 3.8 |
|  | Y17(CG) | α1 | W14(CH2) |  |  |  |  | 3.6 |
|  | Y17(CZ) | α4 | M63(CG) |  |  |  |  | 3.6 |
|  |  | α5 | I72(CG2) |  |  |  |  | 3.7 |
|  | Y17(OH) |  | N73(OD1) |  |  |  |  | 2.7 |
|  | Y17(CD2) |  | Y76(CG) |  |  |  |  | 3.7 |
|  | K20(CE) |  | E79(CD) |  |  |  |  | 3.4 |
|  | L21(CA) |  | I72(CG2) |  |  |  |  | 3.9 |
|  | E24(OE2) |  | S75(OG) |  |  |  |  | 2.6 |
| α3 | N30(CG) |  |  | α10 | K230(CE) |  |  | 3.5 |
|  | N30(OD1) |  |  |  | D234(OD1) |  |  | 3.9 |
|  | Y33(CE2) |  |  |  | Y237(CD2) |  |  | 3.8 |
|  | Y33(OH) |  |  | C-terminal | T243(OG1) |  |  | 2.8 |
|  | L35(CD1) | α5 | F69(CZ) |  |  |  |  | 3.5 |
|  | I37(CD1) |  |  |  | T243(CG2) |  |  | 3.8 |
|  | I37(CG2) |  |  |  | Y241(CE2) |  |  | 3.3 |
|  |  |  |  |  |  | ⍺7-⍺8 connection | L174(CD2) | 3.6 |
|  | K39(CE) |  | MSE63(CA) |  |  |  |  | 3.8 |
|  | K39(CB) |  | F69(CZ) |  |  |  |  | 3.6 |
|  | L40(CD2) |  |  |  | Y241(CB) |  |  | 3.6 |
|  |  |  |  |  | T243(CG2) |  |  | 3.9 |
|  | A41(CB) |  |  |  | Y241(CE1) |  |  | 3.7 |
|  | A41(O) |  |  | α8 | N211(OD1) |  |  | 3.8 |
|  | A41(CB) |  |  |  |  |  | L174(CD1) | 3.5 |
|  | E42(OE1) |  | E66(OE1) |  |  |  |  | 3.2 |
| α4 | H44(CE1) |  |  |  | K209(C) |  |  | 3.6 |
|  |  |  |  |  | Y210(C) |  |  | 3.5 |

**Table S10. Stl^SaPI1^-like repressors**

| **Name** | **Strain** | **Protein accession number** | **Gen accession number (Genomic location)** | **Protein sequence identity (%)** |
| --- | --- | --- | --- | --- |
| Stl^SaPI1^-like SaPI | *Staphylococcus aureus* NTCT9546 | WP_115208074.1 | NZ_UHAK01000002.1  (417268-417987) | 33.49 |
| Stl^SaPI1^-like ShPI | *Staphylococcus hominis FDAARGOS* | SIH39397.1 | FSLI01000015.1  (137382-137948) | 32.65 |
| Stl^SaPI1^-like V.pan | *Virgibacillus panthothenticus* | WP_077296842.1 | NZ_Lt727679.1  (439288-439887) | 31.63 |
| Stl^SaPI1^-like B.sp | *Bacillus sp*. X1 | WP_144546869.1 | NZ_VECT01000031.1  (61128-61724) | 31.28 |
| Stl^SaPI1^-like V.sp | *Virgibacillus sp. SK37 DE0373* | WP_145524117.1 | NZ_VDZE01000056.1  (8525-9187) | 26.39 |
| Stl^SaPI1^-like B.cana | *Bacillus canaveralius ATCC29669* | WP_101577990.1 | NZ_PGVA01000029.1  (45243-45830) | 30.69 |
| Stl^SaPI1^-like B.encl | *Bacillus enclensis CARE-V7 29* | MBH9968881.1 | NZ_JADZLX100000029.1 (12298-12888) | 35.26 |
